# A Kinetic Platform to Determine the Fate of Hydrogen Peroxide in *Escherichia coli*

**DOI:** 10.1371/journal.pcbi.1004562

**Published:** 2015-11-06

**Authors:** Kristin J. Adolfsen, Mark P. Brynildsen

**Affiliations:** Department of Chemical and Biological Engineering, Princeton University, Princeton, New Jersey, United States of America; University of Illinois at Urbana-Champaign, UNITED STATES

## Abstract

Hydrogen peroxide (H_2_O_2_) is used by phagocytic cells of the innate immune response to kill engulfed bacteria. H_2_O_2_ diffuses freely into bacteria, where it can wreak havoc on sensitive biomolecules if it is not rapidly detoxified. Accordingly, bacteria have evolved numerous systems to defend themselves against H_2_O_2_, and the importance of these systems to pathogenesis has been substantiated by the many bacteria that require them to establish or sustain infections. The kinetic competition for H_2_O_2_ within bacteria is complex, which suggests that quantitative models will improve interpretation and prediction of network behavior. To date, such models have been of limited scope, and this inspired us to construct a quantitative, systems-level model of H_2_O_2_ detoxification in *Escherichia coli* that includes detoxification enzymes, H_2_O_2_-dependent transcriptional regulation, enzyme degradation, the Fenton reaction and damage caused by •OH, oxidation of biomolecules by H_2_O_2_, and repair processes. After using an iterative computational and experimental procedure to train the model, we leveraged it to predict how H_2_O_2_ detoxification would change in response to an environmental perturbation that pathogens encounter within host phagosomes, carbon source deprivation, which leads to translational inhibition and limited availability of NADH. We found that the model accurately predicted that NADH depletion would delay clearance at low H_2_O_2_ concentrations and that detoxification at higher concentrations would resemble that of carbon-replete conditions. These results suggest that protein synthesis during bolus H_2_O_2_ stress does not affect clearance dynamics and that access to catabolites only matters at low H_2_O_2_ concentrations. We anticipate that this model will serve as a computational tool for the quantitative exploration and dissection of oxidative stress in bacteria, and that the model and methods used to develop it will provide important templates for the generation of comparable models for other bacterial species.

## Author Summary

Bacterial hydrogen peroxide (H_2_O_2_) response networks contain essential virulence factors for a number of pathogens. Without these systems, infecting bacteria fall prey to host immune cells and cannot establish or sustain an infection. The reaction networks and regulatory features involved are complex, which suggests that computational modeling would facilitate quantitative dissection and analysis of these systems. However, current models of H_2_O_2_ reaction networks have been of limited scope. Here, we constructed a systems-level H_2_O_2_ detoxification model for *Escherichia coli*, and used it to understand how the network responds to different H_2_O_2_ concentrations and insults. We anticipate that this model and comparable ones for other species that are facilitated by its construction will be useful in identifying and understanding methods to sensitize pathogens to immune attack. Such strategies hold great promise for the development of next generation antibiotics, since agents that impair oxidative stress defense systems would focus selective pressure to infection sites, and therefore exhibit slow resistance development and little impact on commensal bacteria.

## Introduction

Reactive oxygen species (ROS) are critical immune antimicrobials used in the first line of defense against infections, where phagocytic cells of the innate immune response use NADPH oxidase to generate an “oxidative burst” of superoxide (O_2_^−^•) after engulfing pathogens in a phagosome [1, 2]. The O_2_•^−^ can then be dismutated to H_2_O_2_ [2], which readily diffuses across the bacterial membrane where it damages sensitive biomolecules, reacts with ferrous iron to produce the highly deleterious •OH [3], or is detoxified by specialized enzymes. The importance of the oxidative burst to immunity is highlighted by the incidence of recurring infections within and shortened life expectancy of patients with defects in NADPH oxidase, a condition known as chronic granulomatous disease (CGD) [4]. In addition, many pathogens including *Bacillus anthracis* [5], *Coxiella burnetti* [6], *Chlamydia trachomatis* (serovars E, K, and L2) [7], *Salmonella enterica* (serovar Typhimurium) [8], *Mycobacterium tuberculosis* [9, 10], *Staphylococcus aureus* [11], *Helicobacter pylori* [12], *Streptococcus pyogenes* [13], and *Enterococcus faecalis* [14] require H_2_O_2_ defense systems to establish or sustain infections. Interestingly, beyond its use by immune cells, bacteria also use H_2_O_2_ against each other, such as when *Streptococcus pneumoniae* stimulates prophage induction and cell death in *Staphylococcus aureus* by generating H_2_O_2_ during niche competitions [15].

Accordingly, bacteria have evolved various pathways to detoxify H_2_O_2_. While the importance of these H_2_O_2_ detoxification systems has been established [16], there are gaps in knowledge regarding the kinetic interplay between them under different conditions. *Escherichia coli* K-12 encodes one alkyl hydroperoxidase (AHP) and two separate catalases for detoxifying H_2_O_2_, which differ in regulation and/or reaction mechanism. AHP and catalase HPI expression are induced by OxyR during oxidative stress, whereas catalase HPII expression is up-regulated in stationary phase and does not increase in the presence of H_2_O_2_ [17–19]. AHP requires one molecule of NADH per reaction cycle, coupling the rate of detoxification achievable by this enzyme to catabolism, whereas H_2_O_2_ is the only substrate in the catalase reaction cycle. AHP has been shown to act as the primary scavenger of endogenously produced H_2_O_2_, and is efficient at detoxifying low concentrations of H_2_O_2_ (<20 μM), whereas catalase is known to dominate clearance at higher concentrations (*>*50 μM) [20, 21]. Since the result of H_2_O_2_ exposure whether that be bacteriostasis, mutagenesis, cell death, or continued growth depends on a kinetic competition for the molecule, it is important to have a quantitative, systems-level understanding of its biochemical reaction network. Due to the complexity of H_2_O_2_ biochemical reaction networks, computational models are necessary for interpretation of H_2_O_2_ detoxification data and prediction of system behavior.

As a result of its importance as a signaling molecule, the most complete models of H_2_O_2_ metabolism currently available were developed for mammalian systems [22–24]. They have included H_2_O_2_ elimination by antioxidants (*e*.*g*., glutathione and thioredoxin) and enzymes (*e*.*g*., catalase, glutathione peroxidase, glutathione reductase, glutaredoxin, and peroxiredoxin), and processing of oxidized protein thiols [22–24]. However, these models were specific to mammalian physiology and did not include transcriptional regulation, enzyme degradation, side reactions of H_2_O_2_ with sensitive biomolecules such as methionine and pyruvate, or the related reactive oxygen species O_2_^−^• and •OH and their associated reactivity (*e*.*g*., •OH rapidly oxidizes all twenty amino acids and glutathione). Although models equivalent to those of mammalian systems have yet to be described for bacteria, there has been progress in modeling subsystems of the H_2_O_2_ response network under H_2_O_2_ stress, such as the thioredoxin system in *E*. *coli* [25].

**Fig 1:**
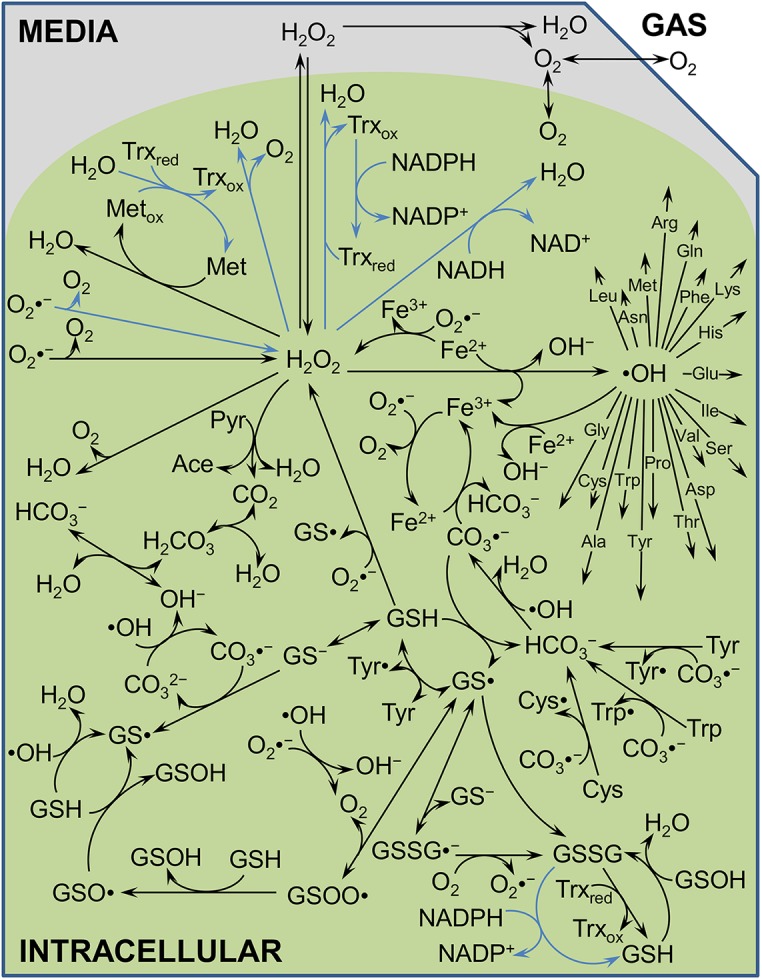
H_2_O_2_ biochemical reaction network. **A.** The kinetic model is separated into three compartments: gas, media, and intracellular. It includes spontaneous reactions (black lines, details in S2 Table) and enzymatic reactions (blue lines, details in S3 Table). Metabolite abbreviations can be found in S1 Table. Information regarding enzyme degradation can be found in S2 Table, and enzyme expression is described in S3 Table. For clarity, •OH reaction products with amino acids and protons are not included in the diagram.

**Fig 2:**
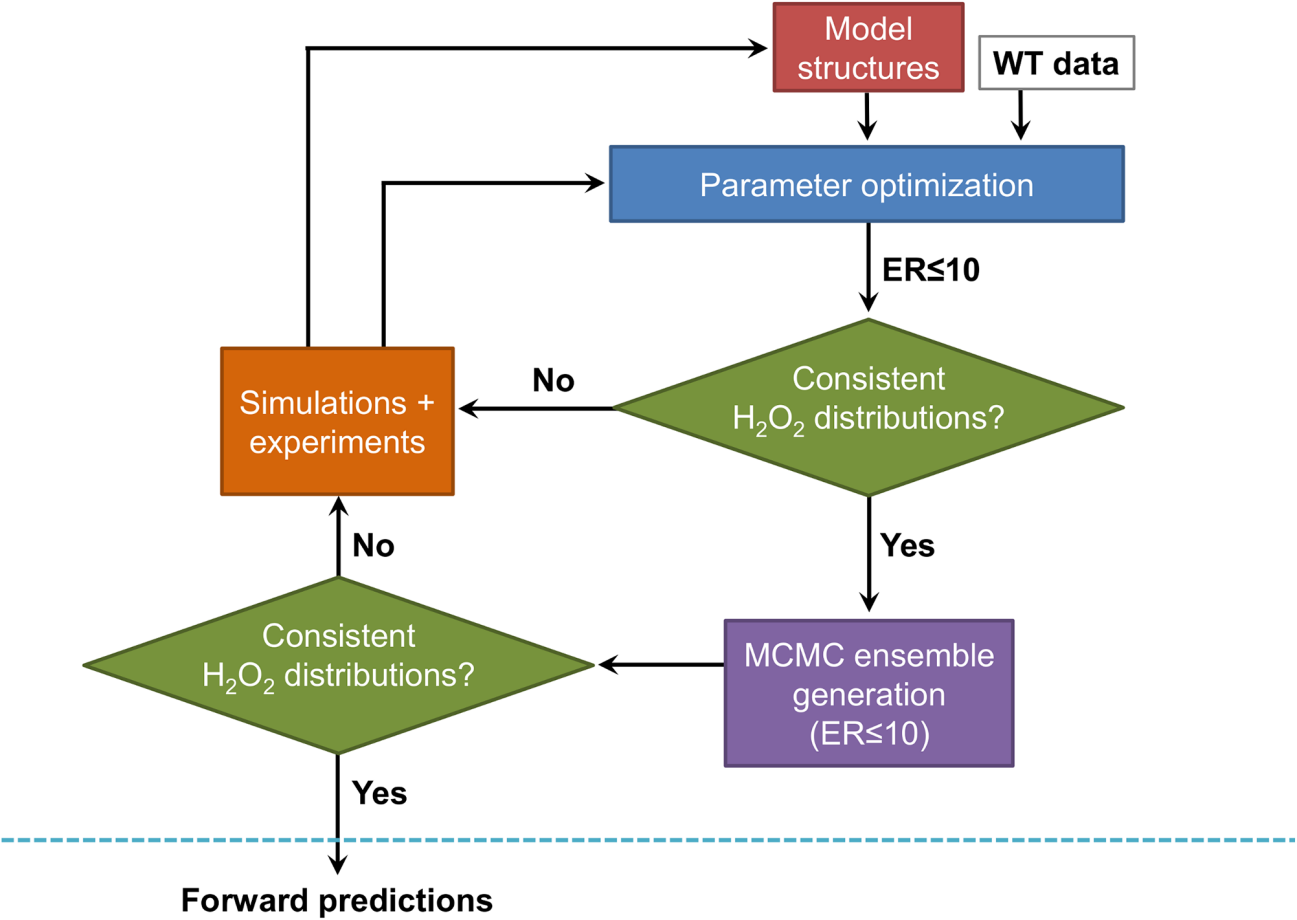
Systematic approach to construct a kinetic model of H_2_O_2_ metabolism. Uncertain parameters in each of the ten model structures are optimized on wild-type clearance data of 10, 25, 100, and 400 μM H_2_O_2_, starting from 1,000 random initial parameter sets. Any models within an evidence ratio of 10 (ER≤10) are used to calculate cumulative H_2_O_2_ clearance by the different detoxification pathways. If the calculated H_2_O_2_ distributions between the models are inconsistent, simulations are used to suggest experiments that differentiate between the disagreeing models, experiments are executed, and the optimization is performed on all experimental data for the model structures that had at least one parameter set with an ER≤10. Once consistent H_2_O_2_ distributions are realized, we identify an ensemble of parameter sets that can all describe the data comparably well with an MCMC procedure. We then assess whether H_2_O_2_ distributions are consistent across the entire ensemble. If the calculated H_2_O_2_ distributions are consistent, we undertake forward predictions.

Here, we have generated a kinetic model of H_2_O_2_ stress in *E*. *coli* whose components are depicted in Fig 1. The biochemical reaction network is compartmentalized into media and intracellular spaces, includes spontaneous and enzymatic detoxification of H_2_O_2_, transcriptional regulation and inactivation of detoxification enzymes, and reactions of H_2_O_2_ and its degradation intermediates (*e*.*g*., •OH) with biomolecules (*e*.*g*., pyruvate, glutathione, all twenty amino acids). Parameters were informed from literature or trained using an iterative and integrated computational and experimental approach (Fig 2). The design criteria we chose to use to develop the model stipulated that consistent discrimination between clearance contributions by the major detoxification systems (AHP, HPI, and HPII) needed to be achieved. Once the design criteria were met, remaining parametric uncertainty was accounted for with use of a Markov chain Monte Carlo (MCMC) procedure to explore the viable parameter space and assemble an ensemble of models that performed comparably well with the training data [26]. The ensemble was then used to quantitatively investigate the importance of carbon availability and translation to H_2_O_2_ detoxification, and its predictions were experimentally confirmed.

## Results

### Design criteria

Our aim was to construct a systems-level kinetic model of H_2_O_2_ detoxification in *E*. *coli* that could provide consistent predictions of H_2_O_2_ distributions among its different detoxification pathways after exposure to a range of initial H_2_O_2_ boluses. To accomplish this goal in the most efficient way possible, we adopted the systematic approach shown in Fig 2. Briefly, we began with a minimal number of experiments, wild-type clearance of different initial H_2_O_2_ concentrations. After optimizing uncertain parameters, we selected models based on their relative likelihood, also referred to as their evidence ratio (ER) [27–32], discarding models more than ten times less likely than the most-likely model in our set (ER≥10). If the acceptable models did not uniformly attribute H_2_O_2_ detoxification to the same pathways, we performed simulations to suggest experiments that could resolve the disagreement. Those experiments were then performed, and data used to arrive at updated parameter estimates. This process was continued until we arrived at a model or set of models that rendered consistent H_2_O_2_ distributions. Since some parameters may not have been important to H_2_O_2_ clearance under the conditions used here, and therefore, unlikely to be informed by the training procedure, we explored the parameter space using a previously developed MCMC procedure [26] to assemble an ensemble of parameter sets that could all describe the H_2_O_2_ clearance data comparably well (ER≤10). In this way, we could ensure that forward predictions were not dependent on ill-defined parameters. We note that this procedure also accounts for cases in which parameter pairings or more complex relationships rather than absolute values are important by varying all parameters simultaneously when walking away from known viable points. Also, before proceeding to forward predictions, we confirmed that all the models within the ensemble still satisfied the design criteria.

### Kinetic model of H_2_O_2_ metabolism in *E*. *coli*

We constructed a compartmentalized reaction network that includes spontaneous and enzymatic reactions present in an *E*. *coli* culture under H_2_O_2_ stress, transcriptional regulation of AHP and HPI, and degradation/inactivation of the major detoxification enzymes AHP, HPI, and HPII. Uncertainty exists with regard to the dynamics of enzyme degradation/inactivation in the presence of H_2_O_2_, as well as the possibility of an H_2_O_2_ gradient across the membrane. Specifically, the enzymes could be degraded or inactivated in an H_2_O_2_-independent manner, either with a fixed degradation constant [33, 34], or optimized to account for the varying degradation rates of different proteins [35, 36]. Alternatively, the H_2_O_2_ detoxification enzymes could be poisoned by their own substrate [37–39], following bimolecular [40] or more complex kinetics [37].

In addition to the indeterminacy in degradation/inactivation kinetics, there is evidence supporting [41] and opposing [42] the presence of an H_2_O_2_ gradient across the cell membrane. Models accounting for these various possibilities are presented in Table 1, along with their corresponding number of uncertain parameters. The introduction of unknown parameters has the potential to improve agreement between model simulations and experimental data solely by increasing the flexibility of the model. For this reason, we calculated the relative likelihood of models, otherwise known as their evidence ratio (ER) [27–32] based on their respective Akaike Information Criterion (AIC), which is a commonly used statistical metric that weighs goodness of fit against model complexity when discriminating between competing models [27, 43–45]. Models with a relative likelihood of ten times less than the best model in the set (ER≤10) were considered acceptable, whereas others were discarded.

**Table 1:** Model types. Rate equations can be found in S2 and S3 Tables, and a more complete description can be found in the Methods.

Structure	AHP deg.	HPI deg.	HPII deg.	Gradient	# Params.
1	Independent, fixed	Independent, fixed	Independent, fixed	No	10
2	Independent, optimized	Independent, optimized	Independent, optimized	No	13
3	Bimolecular	Bimolecular	Bimolecular	No	13
4	Independent, optimized	Complex	Complex	No	13
5	Bimolecular	Complex	Complex	No	13
6	Independent, fixed	Independent, fixed	Independent, fixed	Yes	11
7	Independent, optimized	Independent, optimized	Independent, optimized	Yes	14
8	Bimolecular	Bimolecular	Bimolecular	Yes	14
9	Independent, optimized	Complex	Complex	Yes	14
10	Bimolecular	Complex	Complex	Yes	14

### Experimentally-driven model refinement and parameter estimation

**Fig 3:**
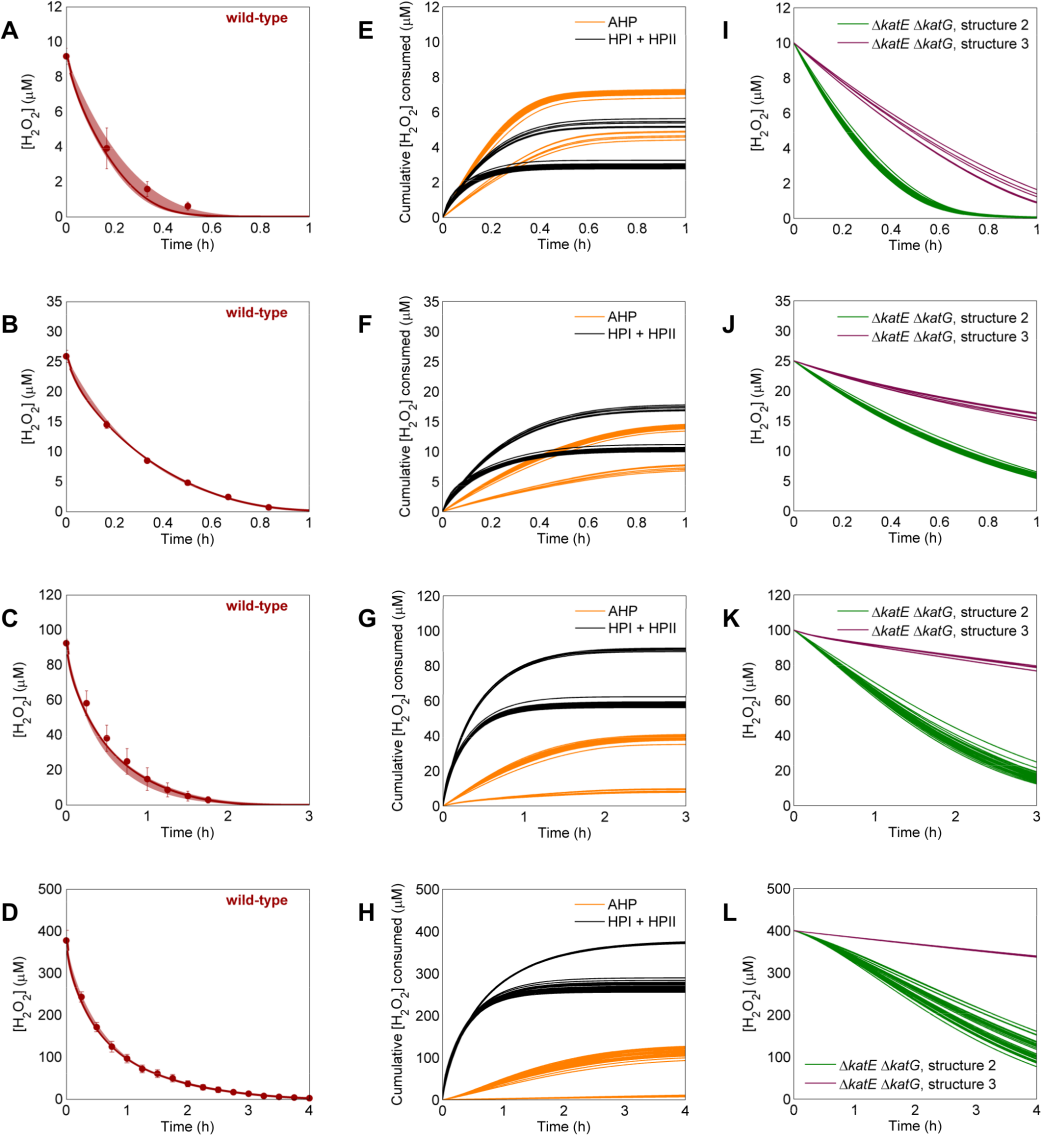
Parameter training on wild-type data and analysis of acceptable models. **A-D**. Parameters for each of the ten different structures were optimized on wild-type clearance of 10 (**A**), 25 (**B**), 100 (**C**), and 400 (**D**) μM H_2_O_2_. Models were ranked using an AIC-based method (Methods), and the 35 models with an ER≤10 were considered viable. Experimental data (solid points) represents at least three biological replicates, with error bars showing the standard error of the mean. Windows represent the maximum and minimum of the 35 acceptable models. Solid lines within the window show the most likely model. **E-H.** Prediction of the amount of H_2_O_2_ cleared by the two major detoxification pathways, AHP (orange) and combined catalase activity (black), after boluses of 10 (**E**), 25 (**F**), 100 (**G**), and 400 (**H**) μM H_2_O_2_. Each line represents the prediction from a single model. **I-L.** Prediction for H_2_O_2_ clearance of 10 (**I**), 25 (**J**), 100 (**K**), and 400 (**L**) μM H_2_O_2_ after removal of all catalase activity (Δ*katE* Δ*katG*). Structure 2 (green) and structure 3 (purple) models predict different clearance dynamics after this perturbation, suggesting that data obtained from this mutant could be used to discriminate between the model structures.

When optimizing parameters simultaneously on clearance of 10, 25, 100, and 400 μM boluses in wild-type cultures, 35 of the 10,000 models had an ER≤10 and were considered viable models. The windows of simulation results of these 35 models are presented in Fig 3A–3D along with the experimental clearance data they were trained on. None of these models contained a gradient; 30 were structure 2 models, and 5 were structure 3. We note that structures that contain a gradient could be favored over those with no gradient under different conditions (*e*.*g*., training on data from a single H_2_O_2_ concentration); however, our goal was to arrive at a model that could describe a wide range of bolus concentrations, and the gain in simulation accuracy for gradient models did not justify addition of the extra parameter as determined by the ER for the experimental conditions considered here. When the H_2_O_2_ distributions of the acceptable models were analyzed, the utility of AHP and the catalases separated into two distinct groups at all bolus concentrations (Fig 3E–3H). The reaction fluxes through AHP and HPI+HPII can be found in S2A–S2D Fig, and those also separated into two distinct groups. We found that these two groups represented predictions made by the two model structures. At all bolus concentrations, structure 2 models predicted a greater contribution by AHP than did structure 3 models. Indeterminacy in catalase null mutant simulations (Fig 3I–3L) suggested that experiments on a strain lacking both HPI (*katG*) and HPII (*katE*) would resolve this discrepancy.

**Fig 4:**
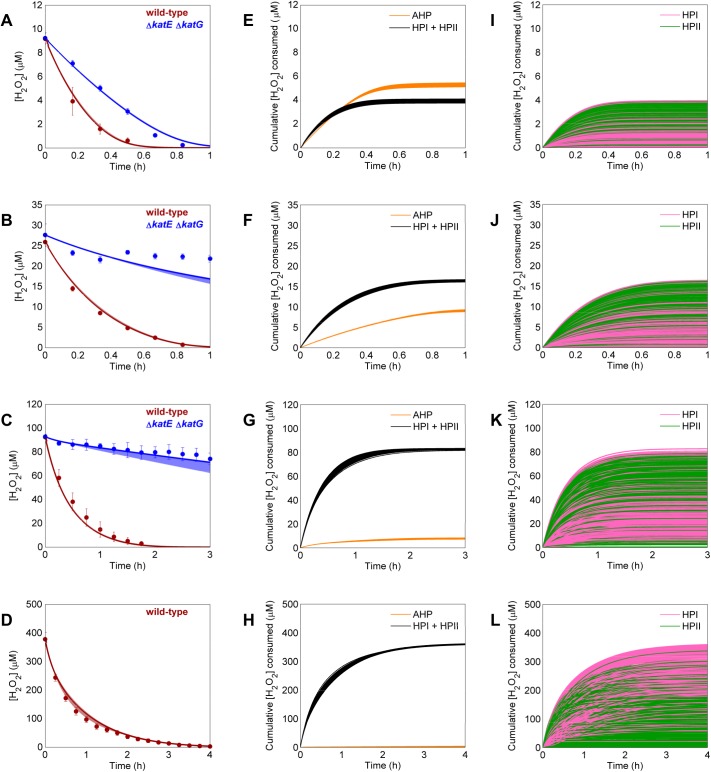
Parameter training on wild-type and Δ*katE* Δ*katG* data and analysis of acceptable models. **A-D**. Parameters for each of the two remaining structures (structures 2 and 3) were optimized simultaneously on clearance of 10 (**A**), 25 (**B**), 100 (**C**), and 400 (**D**) μM H_2_O_2_ by wild-type (red) and a Δ*katE* Δ*katG* mutant (blue). The clearance of 400 μM H_2_O_2_ by Δ*katE* Δ*katG* was omitted from the training procedure because significant cell death was observed (S1H Fig). Experimental data (solid points) represents at least three biological replicates, with error bars showing the standard error of the mean. Windows represent the maximum and minimum of the fits from the 965 acceptable models. Solid lines within the window show the most likely model. **E-H.** Prediction for the amount of H_2_O_2_ cleared by the two major detoxification pathways AHP (orange) and combined catalase activity (black) after boluses of 10 (**E**), 25 (**F**), 100 (**G**), and 400 (**H**) μM H_2_O_2_. Each line represents the prediction from a single model. **I-L.** Prediction for the amount of H_2_O_2_ cleared by the individual catalases HPI (pink) and HPII (green) after boluses of 10 (**I**), 25 (**J**), 100 (**K**), and 400 (**L**) μM H_2_O_2_. Each line represents the prediction from a single model.

Simultaneous training of models on wild-type and Δ*katE* Δ*katG* clearance data was able to resolve the uncertainty between structures 2 and 3. This training iteration resulted in 965 models that all had an ER≤10 (Fig 4A–4D), and all acceptable models were structure 3, which suggests that bimolecular H_2_O_2_-dependent enzyme degradation is an important feature of the detoxification network. We note that clearance of 400 μM H_2_O_2_ by Δ*katE* Δ*katG* was omitted because significant cell death was observed (S1H Fig), and the models were not designed to simulate cell death and possible lysis. All models predicted similar distributions across the major pathways (Fig 4E–4H), but diverged when we looked more closely at the individual catalase contributions (Fig 4I–4L). Reaction fluxes through the major pathways and individual catalases can be found in S2E–S2H Fig and S3A–S3D Fig, respectively. The different parameter sets predicted a range of clearance profiles after removal of either catalase (S4 Fig), suggesting that data obtained from these mutants would resolve the disagreement between models.

**Fig 5:**
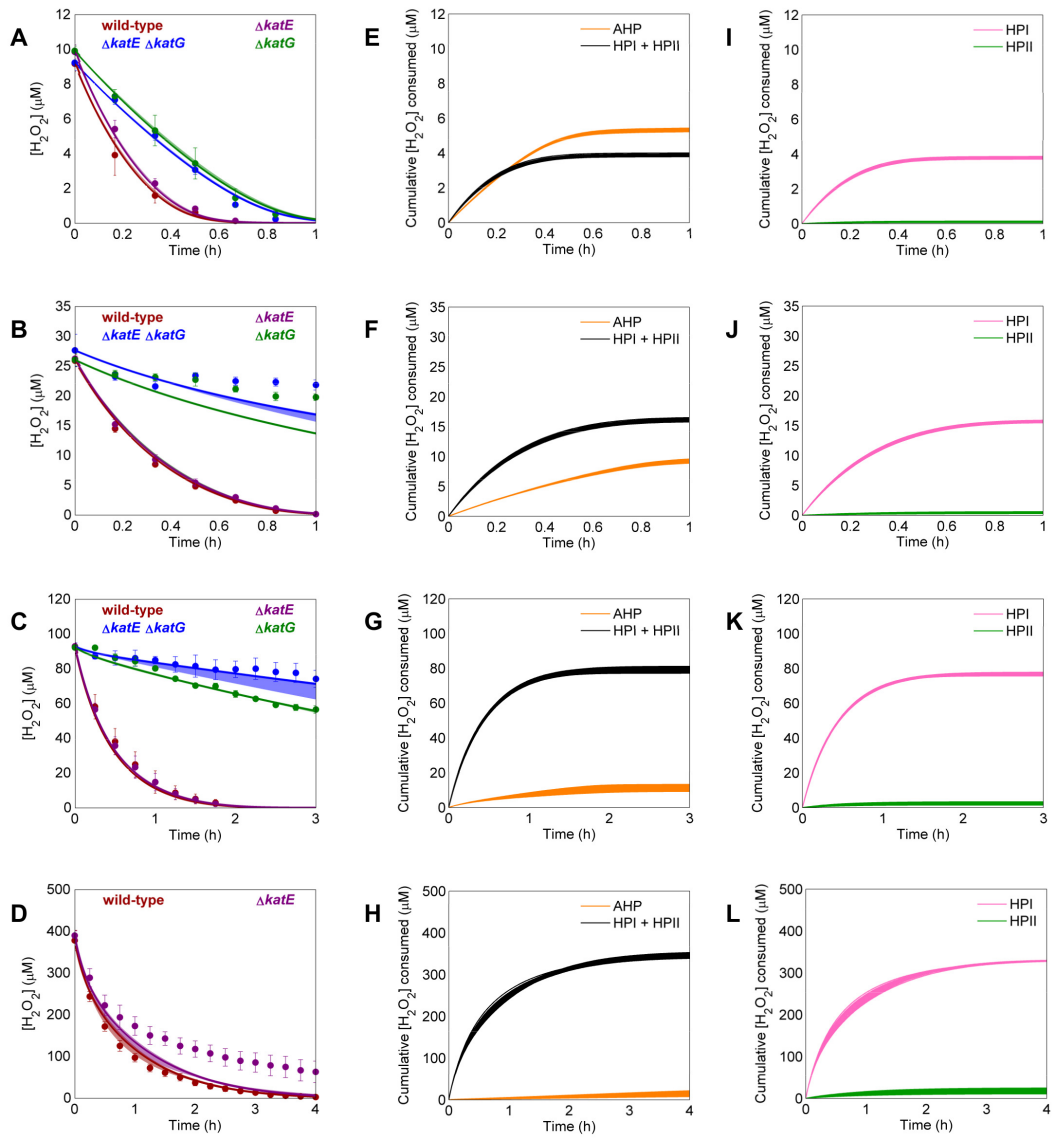
Model training on wild-type, Δ*katE* Δ*katG*, Δ*katE*, and Δ*katG* data and analysis of acceptable models. **A-D**. Parameters for the one remaining structure were optimized simultaneously on clearance of 10 (**A**), 25 (**B**), 100 (**C**), and 400 (**D**) μM H_2_O_2_ by wild-type (red), Δ*katE* Δ*katG* (blue), Δ*katE* (purple), and Δ*katG* (green) data. The clearance of 400 μM H_2_O_2_ by Δ*katE* Δ*katG* and Δ*katG* were omitted from the training procedure because significant cell death was observed (S1H, S1P Fig). Experimental data (solid points) represents at least three biological replicates, with error bars showing the standard error of the mean. Windows represent the maximum and minimum of the fits from the 40 acceptable models. Solid lines within the window show the most likely model. **E-H.** Prediction for the amount of H_2_O_2_ cleared by the two major detoxification pathways AHP (orange) and combined catalase activity (black) after boluses of 10 (**E**), 25 (**F**), 100 (**G**), and 400 (**H**) μM H_2_O_2_. Each line represents the prediction from a single model. **I-L.** Prediction for the amount of H_2_O_2_ cleared by the individual catalases HPI (pink) and HPII (green) after boluses of 10 (**I**), 25 (**J**), 100 (**K**), and 400 (**L**) μM H_2_O_2_. Each line represents the prediction from a single model.

Training uncertain model parameters on wild-type, Δ*katE* Δ*katG*, Δ*katE*, and Δ*katG* data resulted in 40 parameters sets from the 1,000 random initializations that were within an ER of 10 (Fig 5A–5D). All of these models agreed regarding how H_2_O_2_ distributes across not only the major pathways (Fig 5E–5H), but also the individual catalases (Fig 5I–5L). Reaction fluxes through the major pathways and individual catalases can be found in S2I–S2L Fig and S3E–S3H Fig, respectively. The consistent distributions satisfied our design criteria, so we proceeded with the generation of an ensemble of viable parameter sets with which to make forward predictions.

### Exploration of the viable parameter space

The identification of universal “sloppiness” in computational biological models [46], meaning many parameters are poorly constrained after fitting on experimental data, led to the development of a number of methods designed to identify ensembles of parameter sets that could comparably describe the data and be used to assess the robustness of forward predictions [26, 46]. Methods such as “brute force” uniform sampling or Gaussian sampling become impossible with increasingly complex models, so computational biologists have turned to the use of Monte Carlo techniques to explore the viable parameter space efficiently (*e*.*g*., HYPERSPACE [26] and SloppyCell [46]). Here, we used a previously developed MCMC method [26] to explore the parameter space, initiating a random walk away from each of the 40 acceptable parameter sets and keeping 100 viable sets with an ER≤10 for each point. This resulted in an ensemble of 4,000 parameter sets that could all capture our experimental observations, and allowed us to assess robustness of our predictions to parametric uncertainty. In addition, before proceeding, we ensured that all models in the ensemble satisfied our design criteria (S5 Fig).

### Minimal H_2_O_2_ detoxification model

Based on the tight predictions that AHP, HPI, and HPII would dominate clearance (Fig 5), we sought to determine the minimal reaction network required to capture all of our data. To do this, we adopted a previously used two-tiered approach that first deletes reactions in a random order, and then re-optimizes uncertain parameters to determine if adjusted parameters would allow deletion of additional reactions [33]. Beginning with the best model in our ensemble and using this method, we determined that 70 out of our 75 reactions could be removed without increasing the ER beyond a threshold of 10. The essential reactions to the network were the major detoxification enzymes (AHP, HPI, and HPII) and degradation of AHP and HPI. In the case of AHP, a drop in active enzyme could indicate degradation, or alternatively a decrease in available NADH, which is held constant during simulation. On the other hand, H_2_O_2_ is the only substrate of catalase, which suggests that the importance of degradation reflects a decrease in concentration of functional enzyme.

### Parametric sensitivity analysis

In addition to identifying reactions in the network that are dispensable to capturing the H_2_O_2_ clearance data presented in Fig 5, we identified those uncertain parameters that influenced the simulations. Using the optimal parameter set, we individually varied each of the 13 optimized parameters within their bounds. Changes to the Fenton reaction rate constant and Fe^2+^ and Fe^3+^ initial concentrations never increased the ER to beyond 10. All other uncertain/trained parameters perturbed simulations to varying degrees, and their impact was quantified and displayed in S6 Fig

### Impact of carbon deprivation on H_2_O_2_ detoxification

With the model developed and an ensemble of viable parameter sets identified, we sought to assess its predictive capabilities on a physiologically-relevant environmental perturbation. There is growing evidence that microbial killing within macrophages is a combined effect of the toxic environment and a scarcity of nutrients [47–49]. We therefore chose to investigate how H_2_O_2_ detoxification changes during carbon starvation. In the absence of an exogenous source of energy and carbon, the abundance of reducing equivalents can fall to limiting levels [50] and energetic processes such as translation can be hampered [51]. These effects could impact the stress response network by limiting AHP activity and inhibiting H_2_O_2_-dependent induction of AHP and HPI. To determine if carbon starvation in the media used here leads to NADH depletion, we directly measured NADH and NAD+ in M9 media cultures with and without glucose and found that a significant reduction in NADH occurred in carbon-starved cultures (S7 Fig). To see if carbon starvation depresses NADH to levels that inhibit enzyme activities, we measured respiration, which is an NADH-driven process, in M9 media in the presence and absence of glucose and found it to be significantly impaired when glucose was omitted (S8 Fig). In addition, to see if a lack of carbon reduces NADH to levels that impair AHP activity, we monitored clearance of 10 μM H_2_O_2_ in a strain with AHP as the lone major detoxification enzyme (Δ*katE* Δ*katG*), and found that omission of glucose completely inhibited H_2_O_2_ clearance in this strain (S9 Fig). In accordance with these results, model predictions indicated that if NADH was not held constant, AHP would drain it from the system in less than a second (S10 Fig). The impact of glucose starvation on induction of AHP and HPI expression was also assessed with the use of GFP reporter plasmids. Omission of glucose completely inhibited H_2_O_2_-dependent induction (S11 Fig). Therefore, to simulate the impact of carbon deprivation, NADH concentrations were no longer held constant and protein production was set to zero.

**Fig 6:**
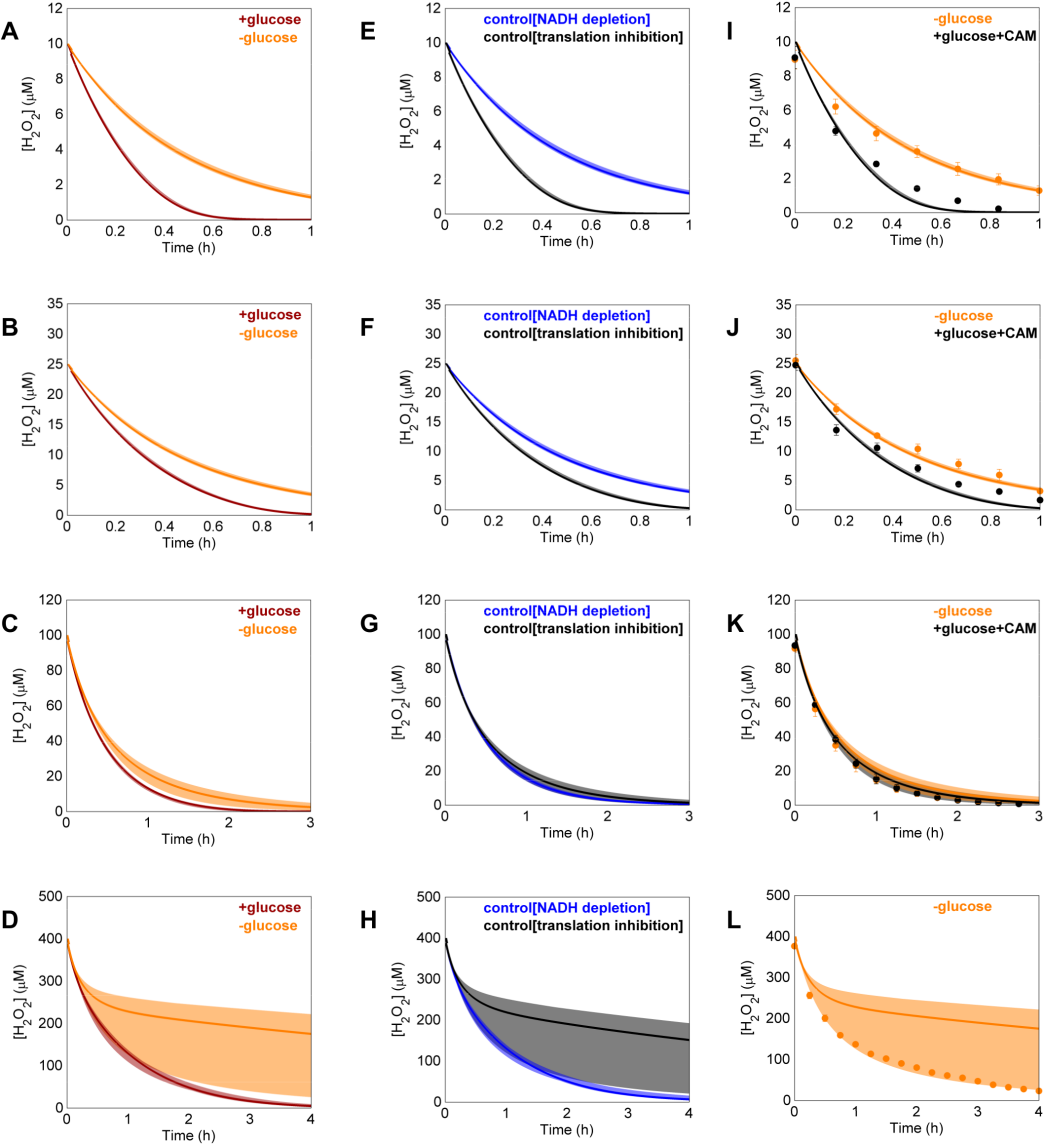
Predictions for clearance during carbon deprivation. **A-D**. Ensemble predictions for H_2_O_2_ clearance of 10 (**A**), 25 (**B**), 100 (**C**), and 400 (**D**) μM H_2_O_2_ by wild-type in M9 10 mM glucose (red) and M9 lacking glucose (orange). The spontaneous rate of H_2_O_2_ degradation differs in media with and without glucose, and this parameter was optimized using cell-free controls and adjusted to reflect the different spontaneous degradation during simulations. **E-H.** Ensemble simulations for H_2_O_2_ clearance of 10 (**E**), 25 (**F**), 100 (**G**), and 400 (**G**) μM H_2_O_2_ by wild-type with only NADH depletion (blue) or translation inhibition (black). Since these were controls for the–glucose predictions, the spontaneous H_2_O_2_ degradation rate matched that of media lacking glucose. **I-L.** Experimental measurement of clearance of 10 (**I**), 25 (**J**), 100 (**K**), and 400 (**L**) μM H_2_O_2_ by glucose-deprived (orange) and CAM-treated (black) cultures, shown with their predicted profiles. Experimental data (solid points) represents three biological replicates, with error bars showing the standard error of the mean. Results for clearance of 400 μM H_2_O_2_ by CAM-treated cultures were omitted based on significant cell death (S1X Fig). In all simulations, windows represent the maximum and minimum of the predictions from the 4,000 models in the ensemble. Solid lines within the window show the most likely model.

Ensemble predictions for carbon deprivation (- glucose) were made using the complete reaction network and are shown in Fig 6A–6D, along with the carbon-replete control (+ glucose). These predictions were experimentally confirmed and the data are presented in Fig 6I–6L, orange). To quantify how the different elements of glucose deprivation (NADH limitation, inhibition of translation) contributed to the observed phenotypes, we investigated the individual effects of NADH depletion or translation inhibition with simulation controls (Fig 6E–6H). At lower treatment concentrations (10 and 25 μM H_2_O_2_), starvation was predicted to slow detoxification as a result of NADH depletion, whereas inhibition of protein synthesis was predicted to have a negligible effect. At 100 μM H_2_O_2_, glucose-deprived cultures were predicted to clear H_2_O_2_ comparably to glucose-fed cultures, with neither reducing equivalent availability nor enzyme production substantially hindering detoxification. The impact of starvation at 400 μM H_2_O_2_ was predicted to be largely mediated by translation. We note that although selective inhibition of NADH production and usage was not feasible due to the wide variety of sources and sinks, targeted inhibition of translation was experimentally tractable with the use chloramphenicol (CAM) (S11 Fig). Experimental confirmation of clearance by CAM-treated cultures is shown in Fig 6I–6K. Unfortunately, CAM-treatment led to cell death at 400 μM H_2_O_2_ (S1X Fig), which prevented direct confirmation of the prediction at that concentration. Interestingly, this cell death suggested that translation of some protein other than AHP or HPI is important to survival at 400 μM H_2_O_2_, because we demonstrated that carbon deprivation inhibited induction of AHP and HPI at 400 μM H_2_O_2_ (S11 Fig), and it is known that carbon deprivation can have promoter-specific effects [51] and CAM stops synthesis of all proteins.

## Discussion

The toxic nature of H_2_O_2_ makes it an ideal weapon in inter-species warfare, and it is used as such by the immune system during infection [2] and even by other bacteria in niche competitions [15]. Bacteria have evolved numerous defense systems, which can differ significantly in their substrate requirements, reaction mechanisms, and regulation, and the complexity of these defense networks and the broad reactivity of H_2_O_2_ necessitate the use of computational modeling for quantitative interpretation and prediction of H_2_O_2_ distributions in cells [52]. Due to its importance as a signaling molecule, models of H_2_O_2_ metabolism in mammalian systems have been constructed, and they have included enzymatic detoxification of H_2_O_2_ [22–24], oxidation of cysteine residues [22], transport of H_2_O_2_ across membranes [22–24], and oxidation of targets involved in signaling [24]. However, beyond their specificity for mammalian systems, none have accounted for uncertainty in optimized parameters or included synthesis or inactivation of enzymes, side reactions of H_2_O_2_, or other reactive oxygen species present in the network. In bacteria, modeling efforts have focused on subsystems affected by H_2_O_2_ stress, such as that of the thioredoxin system in *E*. *coli*, which included the oxidation of thioredoxin and the reduction of oxidized thioredoxin, methionine sulfoxide, protein disulfides, and 3’-phosphoadenosine-5’-phosphosulfate [25]. These previous efforts inspired us to construct a quantitative, systems-level model of H_2_O_2_ stress in *E*. *coli* that includes media and cellular compartment-specific species and reactions; H_2_O_2_-dependent transcriptional regulation, inactivation, and activity of H_2_O_2_ detoxification enzymes; reductases to reduce oxidized species; O_2_^−^• and •OH and their related reactions (*e*.*g*., oxidation of all twenty amino acids by •OH); and reactions of H_2_O_2_ with other metabolites such as glutathione and the α-keto acid pyruvate. In addition, we addressed structural uncertainty in the model using an iterative computational and experimental methodology, and assessed parametric uncertainty using an MCMC procedure, which enabled the robustness of model predictions to be assessed. Similar ensemble approaches have become popular methods to account for parametric uncertainty [53–62], and several techniques have been developed to efficiently explore parameter spaces [26, 46].

One power of quantitative computational modeling is its ability to predict emergent systems behavior [33, 44, 63–65]. For instance, Schaber and colleagues used an ensemble of possible models describing different hypotheses regarding the mechanism of the high osmolarity glycerol (HOG) pathway in yeast to uncover novel features of the pathway [44]. Here, we leveraged our model to gain a quantitative understanding of how carbon deprivation, which bacteria encounter in phagocytes [47–49], affects H_2_O_2_ detoxification by *E*. *coli*. Accounting for the NADH limitation and translational inhibition that occurs with carbon source starvation, our simulations were able to correctly predict H_2_O_2_ clearance dynamics. Upon dissection of simulation results, delayed clearance at lower concentrations was attributed to reduced AHP activity from NADH depletion, whereas at higher concentrations carbon-starved cultures resembled carbon-replete cultures because pre-expressed HPI dominated H_2_O_2_ detoxification, suggesting that both NADH availability and induction of AHP and HPI synthesis at H_2_O_2_ concentrations > 25μM were of minor importance. We note that changes in concentrations of other metabolites, such as ATP, occur in carbon-starved cultures [66], and that they were not explicitly accounted for here because they did not directly act as a substrate in any of the reactions of the model. Rather we anticipate that some of the metabolite perturbations were implicitly accounted for because they contributed to the inhibition of translation and/or depletion of NADH that were included. These data demonstrate that the nutritional status of the environment can have a major impact on bacterial H_2_O_2_ defenses, but the extent of that impact depends on the quantitative level of H_2_O_2_. Beyond carbon deprivation, it would be interesting to see how other types of starvation (*e*.*g*., sulfate, iron) influence H_2_O_2_ detoxification, since bacteria are subjected to oxidative stress in various scenarios [1, 15, 67], and we expect that dependencies distinct from those of carbon source starvation could be observed if other types of limitation influence NADH availability and translation differently.

In immune cells, phagocytized bacteria are exposed to ROS with an oxidative “burst” from NADPH oxidase, which then tapers over time [68–70]. In this work, we examined detoxification of a burst of H_2_O_2_ with bolus treatments, and note that more complex treatment dynamics could be handled by the model developed here. For instance, the model could be adjusted for continuous treatment by adding an H_2_O_2_ delivery reaction, which could be achieved experimentally with a fed-batch reactor. Alternatively, H_2_O_2_ could be provided through indirect means, such as with exposure to redox-cycling agents like paraquat [71]; though obtaining accurate estimates of H_2_O_2_ production from such compounds could be a challenge, because generation would be cell-dependent. Different H_2_O_2_ delivery dynamics could have a profound impact on the kinetic competition for H_2_O_2_, and as long as H_2_O_2_ influx can be accurately accounted for the model developed could prove invaluable for interrogating its distribution.

One area of growth for the platform we developed is adaptation of the model to allow for analysis of lethal H_2_O_2_ concentrations. In its current form, the size of the cellular compartment is fixed and enzymatic reactions do not occur in the media compartment. To model lethal concentrations of H_2_O_2_, cell lysis has to be accounted for in terms of reduction in the volume and surface area of the cellular compartment and the addition of certain enzymatic activities to the media compartment, such as catalase, which can function when released from cells. In addition, it might be necessary to diversify the cellular compartment if all cells that die do not lyse but contain compromised translational and/or catabolic activities. Despite this added complexity, the ability to accurately simulate lethal damage, such as that involving DNA and the membrane, could provide insight into H_2_O_2_-induced death.

Models akin to the one described here will improve understanding of bacterial defenses against host immune responses, and possibly suggest targets for novel anti-virulence therapies [52]. For example, an existing model of nitric oxide (NO•) defenses in *E*. *coli* [33] has provided valuable predictions regarding NO• delivery rates that maximize antimicrobial activity [63]. Additionally, it provided a framework that allowed for a model-guided investigation of the underlying mechanism of an NO•-sensitizing mutation, ΔclpP, in *E*. *coli* [72]. The success of the NO• model provided inspiration to develop a similar model for H_2_O_2_, which is another toxic, diffusible metabolite used by immune cells when fighting infection [1, 2]. We anticipate that the H_2_O_2_ model developed here will yield novel quantitative insight into the kinetic competition for H_2_O_2_ in *E*. *coli* and provide a framework for the mechanistic investigation of perturbations that affect clearance, while illuminating targets to sensitize bacteria to immune attack.

## Materials and Methods

### Bacterial strains

*E*. *coli* K-12 MG1655 was used in all experiments. Δ*katE* and Δ*katG* mutations were transduced into MG1655 from their respective strains in the Keio collection [73] by the P1 phage method. The Δ*ahpCF* mutation was generously provided by Michael Kohanski and transduced into MG1655 using the P1 phage method. All antibiotic markers were cured out using pCP20 [74]. The known antioxidant pyruvate (25 mM) was included in all LB agar plates to prevent a toxic build-up of H_2_O_2_ [75]. Deletions were PCR checked for proper chromosomal integration with a forward primer external to the gene and reverse primer within the kanamycin resistance cassette (kan^R^) before curing. Internal primers were used to check for gene duplication. In cured strains, external primers before and after the gene were used to check for proper scar size. All PCR primers are listed in S4 Table.

### Measurement of H_2_O_2_ clearance

Overnight cultures were inoculated from −80°C stocks and grown for 20 hours in 1 mL LB + 30–75 U/mL catalase (bovine liver catalase at 2,000–5,000 units/mg protein: Sigma Aldrich), then used to inoculate 20 mL M9 10 mM glucose medium + 30–75 U/mL catalase to an OD600 of 0.01 in 250 mL baffled flasks. Catalase was added to prevent the possibility of H_2_O_2_ accumulation in strains lacking major detoxifying enzymes, and added to wild-type cultures to maintain consistency across strains. The catalase concentration was chosen based on the amount required to maintain growth in a mutant lacking all major detoxification enzymes (Δ*katE* Δ*katG* Δ*ahpCF*), beyond which increasing the catalase concentration no longer increased growth rate or terminal cell density in the case of overnights. Cultures were grown at 37°C with shaking at 250 rpm for 8 h (OD_600_ 0.3–0.6, 0.15 for the slower growing Δ*katE* Δ*katG* Δ*ahpCF*). After the 8 hour growth period, 12 mL of culture was removed to a pre-warmed 15 mL Falcon tube and centrifuged at 37°C and 4,000 rpm for 10 min. 10.8 mL of spent media was removed, the cell pellet resuspended, and 1 mL transferred to a warm 1.5 mL microcentrifuge tube. Cells were washed a total of four times to remove all catalase. Washes consisted of spinning down at 14,000 rpm for 2 min, removing 980 μL of media, and resuspending the cell pellet with 980 μL fresh warm media. For samples lacking glucose during challenge with H_2_O_2_, glucose was omitted during the final wash step and in the inoculated flask. For CAM treatment assays, all wash steps were performed with 100 μg/mL CAM.

Prior to inoculation with washed cells, 20 mL fresh M9 10 mM glucose media in 250 mL baffled flasks were warmed to 37°C. A bolus of 10, 25, 100, or 400 μM H_2_O_2_ was added to the flasks, and the time 0 point was measured, after which flasks were inoculated to an OD_600_ of 0.01. At desired time points, 200 μL was removed to a 1.5 mL microcentrifuge tube and centrifuged at 15,000 for 3 min. 150 μL of the supernatant was moved to a sterile microcentrifuge tube and stored at 4°C until H_2_O_2_ concentration could be measured. Samples were assayed for H_2_O_2_ within 2 h of harvesting. H_2_O_2_ in the supernatant was measured using the Amplex Red Hydrogen Peroxide/Peroxidase kit (Life Technologies) according to the manufacturer’s instructions after dilution to below 10 μM H_2_O_2_. A standard curve spanning 0 to 10 μM H_2_O_2_ was used to calculate H_2_O_2_ concentrations. A fresh standard curve was produced for each Amplex Red assay to account for increasing background fluorescence over the course of the day due to the sensitivity of Amplex Red to both light and air [76].

To assess whether centrifuging to remove cells was sufficient, we compared values from this method to sterile filtering (0.22 μm pore size) of samples, as well as centrifuging + filtering, for the 30 min point in the 400 μM H_2_O_2_ clearance assay (S12 Fig). Centrifuging alone was not significantly different from filtering (p = 0.45) or centrifuging + filtering (p = 0.40) based on a two-sample t-test with unequal variance. To determine if the exogenous catalase that was added to the pre-culture steps affected clearance profiles, we performed identical experiments for wild-type propagated without catalase in all steps (S13 Fig). The presence of catalase in the pre-culture did not affect wild-type clearance of H_2_O_2_ at any concentration.

### Measurement of colony forming units (CFUs)

To determine whether H_2_O_2_ treatment resulted in cell death, we quantified CFUs throughout the clearance assays. After isolating the H_2_O_2_-containing supernatant for Amplex Red assays as described above, an additional 30 μL supernatant was removed from centrifuged samples and discarded, to achieve a greater fold-dilution of H_2_O_2_ during the first wash step. In the first wash step, 980 μL of PBS was added and the cell pellet was resuspended. Samples were centrifuged again at 15,000 rpm for 3 min, 980 μL of the supernatant was removed, and the cell pellet was resuspended a final time in 80 μL PBS. Plating was performed using the serial dilution method, and samples were plated on LB agar containing 25 mM pyruvate to scavenge any residual H_2_O_2_ remaining in the pellet and any endogenously produced H_2_O_2_ in scavenging-deficient strains. Plates were incubated for 16 h at 37°C prior to counting colonies.

### Oxygen measurement

MG1655 cultures were grown and washed identically to the H_2_O_2_ clearance assay. After the final wash, the resuspended cells were used to inoculate 10 mL of pre-warmed M9 with or without glucose in a 50 mL Falcon tube containing a sterile magnetic stirring bar, immersed in a stirred water bath at 37°C, to an OD_600_ of 0.1. Cells were allowed to consume oxygen for ten minutes before being treated with 5 mM KCN to halt respiration, which consumes the majority of O_2_ in *E*. *coli* cell cultures under these conditions [63]. The percent oxygen saturation was measured at a frequency of one reading per second using the FireStingO2 fiber-optic O2 meter with the OXROB10-CL2 robust oxygen miniprobe (PyroScience, GmbH). Temperature fluctuations were compensated for using the TDIP15 temperature sensor (PyroScience GmbH) and the FireSting Logger Software. The equilibrium oxygen concentration was used to convert the percent saturation to concentration, and was determined by calibrating the probe in ultrapure Milli-Q water at 37°C, which has an oxygen concentration of 210 μM [77], and transferring the probe to air-saturated M9 media. The equilibrium concentration of the media matched that of the ultrapure water.

### NAD+/NADH measurement

Overnight cultures were inoculated and grown identically to the H_2_O_2_ clearance assay, and used to inoculate 20 mL M9 10 mM glucose medium + 30–75 U/mL catalase to an OD600 of 0.01 in 250 mL baffled flasks. Cultures were grown at 37°C with shaking at 250 rpm to an OD_600_ of 0.2 (~6.5 h). Four 1 mL aliquots were transferred from the flask to warm, 1.5 mL microcentrifuge tubes and centrifuged at 15,000 rpm for 3 min. The media was removed, and the pellets were resuspended with 1 mL fresh M9 with or without 10 mM glucose and transferred to warm test tubes. The tubes were then incubated at 37°C with shaking at 250 rpm for 60 min. The time 0− point was taken directly from the flask prior to centrifuging and resuspension.

NAD+ and NADH were measured using the EnzyChrom NAD/NADH Assay Kit (BioAssay Systems) following the manufacturer’s protocol, except for a brief sonication step. For each measurement, 400 μL (NAD+) or 800 μL (NADH) of cell culture was transferred from the flask (time 0) or test tube (60 min) to a 1.5 mL microcentrifuge tube. The tubes were centrifuged for 3 min at 15,000 rpm, and 380 μL (NAD+) or 780 μL (NADH) of supernatant was removed and discarded. The cell pellets were resuspended in 100 μL of either NAD or NADH extraction buffer and sonicated for 20 s at room temperature at an amplitude of 10 using a Fisher Scientific Model 50 Sonic Dismembrator. The extracts were heated at 60°C for 5 min, before adding 20 μL of assay buffer and 100 μL of the opposite extraction buffer. Samples were then vortexed briefly and centrifuged for 5 min at 14,000 rpm. The supernatants were used for the NAD/NADH assay following manufacturer’s instructions. An NAD+ standard curve from 0–2 μM was generated each day and used to convert absorbance to concentration. The standard curve underwent extraction protocols identical to cell samples, including sonication and heating. Since NAD+ and NADH produce identical standard curves, and NADH is more unstable, only NAD+ was provided in the kit and was used to convert both NAD+ and NADH absorbance to concentration.

### Reporter assays

MG1655 was transformed with pUA66 P_*ahpC*_ *-gfp*, pUA66 P_*katG*_ *-gfp*, and pUA66 P_*katE*_ *-gfp*, which were all obtained from a pre-existing library [78]. Overnight cultures were inoculated from −80°C stocks and grown for 20 h in 1 mL LB + 30–75 U/mL catalase + 30 μg/ml kanamycin for plasmid retention, then used to inoculate 20 mL M9 10 mM glucose medium + 30–75 U/mL catalase + 30 μg/ml kanamycin to an OD_600_ of 0.01 in 250 mL baffled flasks. Cultures were grown, washed, and treated with H_2_O_2_ identically to the protocol described in the Amplex Red assay protocol. Washed cells were fixed before inoculation to the H_2_O_2_-containing flasks to provide a time 0− sample for each condition. Final points were sampled and fixed when ~90% of the H_2_O_2_ had been cleared by wild-type in 10 mM glucose M9 media: 30 min for the 10 μM H_2_O_2_ flask, 40 min for the 25 μM H_2_O_2_ flask, 1 h 15 min for the 100 μM H_2_O_2_ flask, and 2 h for the 400 μM H_2_O_2_ samples. Fixing involved removing 1 mL culture to a microcentrifuge tube and centrifuging at 15,000 rpm for 3 min, removing 980 μL supernatant, and resuspending with 480 μL 4% paraformaldehyde (PFA). After 25 min at room temperature, the samples were again centrifuged at 15,000 rpm for 3 min, 480 μL of the PFA was removed, and the pellet was resuspended with 980 μL 1X PBS. Samples were stored at 4°C until analysis by flow cytometry on an LSR II flow cytometer (BD Biosciences, San Jose, CA), where green fluorescence was measured on a per cell basis. Fluorescence was measured using 488 nm excitation and a 525/20 bandpass filter, and data were acquired using FACSDiVa software (BD Biosciences, San Jose, CA).

### Model framework

The modeling framework used in this work largely followed that used by Robinson and Brynildsen [33]. It is composed of a system of ordinary differential equations that are numerically integrated to provide predicted species concentration over time. We begin with a mole balance for all species in the model:

$$\frac{dN}{dt}=S\cdot v$$ (1)

where ***N*** is an *s* x 1 vector representing the total amount of given species in moles, ***S*** is the *s* x *r* stoichiometric matrix, and ***v*** is an *r* x 1 vector representing reaction rates in moles per time. Here, *s* indicates the number of species in the model, and *r* indicates the number of reactions in the model. Since most reaction rates are calculated on the basis of concentration per time, the rate vector was converted into these units in the following manner.

$$\frac{dN}{dt}=S\cdot V_{\mathrm{rxn}}\cdot r$$ (2)

where ***V***_***rxn***_ is an *r* x *r* diagonal matrix representing the volumes of the compartments in which the reactions are taking place: *V*_*cell*_ for intracellular reactions, *V*_*media*_ for reactions taking place only in the media, and *V*_*total*_ for exchange reactions. Due to experiments also being performed on a concentration basis, *N* was converted into units of concentration by performing the following operation.

$$V_{\mathrm{spec}}^{-1}\frac{dN}{dt}=V_{\mathrm{spec}}^{-1}\cdot S\cdot V_{\mathrm{rxn}}\cdot r$$ (3)

where ***V***_***spec***_ is a diagonal *s* x *s* matrix of species compartment volumes: *V*_*cell*_ for intracellular species, *V*_*media*_ for species in the media compartment, and *V*_*total*_ for species that freely diffuse across the cell membrane (*e*.*g*., O_2_, H_2_O_2_ in non-gradient models). The left-hand side of the equation is equivalent to *d* ***C*** */dt* when the volume does not vary appreciably over the course of the experiment. To avoid having a culture-volume-specific model, we transformed the volume dependencies into volume fractions by multiplying and dividing by V_total_ (*V*_*cell*_ */V*_*total*_, *V*_*media*_ */V*_*total*_, *V*_*total*_ */V*_*total*_) and rearranging the equation with the use of the commutative property of scalar multiplication.

$$\frac{dC}{dt}=\frac{V_{total}}{V_{total}}\cdot V_{\mathrm{spec}}^{-1}\cdot S\cdot V_{\mathrm{rxn}}\cdot r$$ (4)

$$\frac{dC}{dt}=V_{total}\cdot V_{\mathrm{spec}}^{-1}\cdot S\cdot \frac{1}{V_{total}}\cdot V_{\mathrm{rxn}}\cdot r$$ (5)

$$\frac{dC}{dt}=F_{\mathrm{spec}}^{-1}\cdot S\cdot F_{\mathrm{rxn}}\cdot r$$ (6)

where ***F***_***spec***_ is an *s* x *s* diagonal matrix of the volume fractions for species and ***F***_***rxn***_ is an *r* x *r* diagonal matrix of the volume fractions for reactions. By making this adjustment, we avoid the need of requiring total culture volume as an input into the model, and simplify the input to optical density (OD_600_) that can readily be converted to volume fractions.

### Initial species concentrations

Most initial species concentrations were obtained from literature (S1 Table)[21, 25, 79–90]. The equilibrium concentration of oxygen was determined by calibrating a FireStingO2 fiber-optic O_2_ meter with the OXROB10-CL2 robust oxygen miniprobe (PyroScience, GmbH) in ultrapure Milli-Q water at 37°C and transferring it to air-saturated M9 10 mM glucose medium at 37°C. We calculated the concentration in our media by comparing it to the known value in deionized water [77], which is 210 μM. The value in our media was equivalent. The initial H_2_O_2_ concentration was set to the initial average value of the data when optimizing parameters (*e*.*g*., 25.89 μM instead of 25 μM for wild-type) to avoid penalizing model fit for experimental error. When making forward predictions, the concentration was set to the anticipated initial concentration (*e*.*g*., exactly 25 μM).

Initial species that were trained on experimental data included AHP, HPI, HPII, Fe^2+^, and Fe^3+^. While experimental measurements on AHP [21], HPI [82], and HPII [82] are available, their concentrations vary with environment and growth phase, as shown in our reporter assays (S11 Fig). AHP, HPI, and HPII are the major H_2_O_2_ detoxification systems in *E*. *coli*. We therefore allowed flexibility in their initial concentrations, constraining them to be within the range of 0–20 μM. Because individual concentrations of Fe^2+^ and Fe^3+^ are unresolved, but experimentally measured to have a combined concentration of 10 μM, we allowed their initial concentrations to vary from 0 to 10 μM.

### HPI and HPII reaction mechanism

Catalase activity follows a ping-pong mechanism, reacting with one H_2_O_2_ to form a reactive intermediate, followed by reaction with a second H_2_O_2_ molecule to return the enzyme to its original form [91, 92]. Given that the substrate in the first and second reaction is the same, the rate equation simplifies to a Michaelis-Menten type structure. While inhibition of catalase activity by H_2_O_2_ becomes apparent at high concentrations (*e*.*g*., greater than 100 mM H_2_O_2_ for *E*. *coli* HPII [92]), it is assumed negligible at the concentrations used in our experiments, and Michaelis-Menten kinetics are appropriate. Rate equations and constants can be found in S3 Table (Reactions 69 and 70). While HPI has the ability to utilize other reducing agents at low H_2_O_2_ concentrations, this activity is significantly slower than its catalase activity (about 1% the k_cat_ of its catalase activity [21]), so the peroxidase activity was assumed to negligibly contribute to H_2_O_2_ detoxification in this study.

### AHP reaction mechanism

The AHP reaction cycle begins when the peroxidatic cysteine of AhpC reacts with H_2_O_2_ to form a sulfenic acid, which resolves to form a disulfide bond with another cysteine residue. The active AhpC is regenerated by its reductase partner AhpF, which uses NADH as an electron donor [38]. We modeled this cycle using ping-pong reaction kinetics, with H_2_O_2_ and NADH as the substrates, a structure which has been used previously [38]. Kinetic parameters were not available for *E*. *coli*, but a protein BLAST search [93] revealed 98% protein sequence identity for AhpC and 95% identity for AhpF between *E*. *coli* MG1655 and *S*. Typhi, so available parameters for *S*. Typhi were used [38, 94]. Additional information and rate constants can be found in S3 Table (Reaction 71).

### Expression of detoxification enzymes

In this work, the main experimental variable was the concentration of H_2_O_2_, and therefore, we opted for simplicity and only H_2_O_2_-dependent regulation of gene expression was considered. The expression of catalase HPI and AHP increase in response to H_2_O_2_ [17, 95], whereas the expression of catalase HPII is not dependent on H_2_O_2_ [17, 18]. These dependencies were confirmed using transcriptional reporters for *ahpC*, *katE*, and *katG*, and measuring fluorescence on a per cell basis using flow cytometry (S11 Fig). Following previous dynamic models, gene expression was modeled using a Hill equation with a coefficient of n = 1 [33, 34], except for HPII which had initial concentration but was not expressed further. In addition, transcription was assumed to be limiting in the production of active enzyme, as assumed previously [33, 34], and the bioavailability of ferroheme *b*, which is an essential cofactor of HPI, was assumed to not be rate limiting. HPI and AHP are expressed according to Reactions 67 and 68, respectively, in S3 Table. The maximum expression rate and Hill equation constants K_AHP-exp,H2O2_ and K_HPI-exp,H2O2_ are informed during parameter optimization. The bounds on the maximum expression rates are based on the highest and lowest maximum expression rates found in the work of Kotte and colleagues [34], and have been used previously when optimizing unknown expression rates [33]. Bounds on K_AHP-exp,H2O2_ and K_HPI-exp,H2O2_ were approximated by the work of Kotte and colleagues [34], which varied from approximately 2 nM to 1 mM. Here, we allowed variation from 0 to 1 mM. We note that while the parsimonious treatment of H_2_O_2_-dependent expression was sufficient in this work, exploration of new environments could necessitate a more comprehensive modeling of transcriptional regulation, since expression of the detoxification enzymes in this work are known to depend on numerous regulators (*e*.*g*., OxyR, RpoS, FIS, Fur) [96].

### Modeling of enzyme degradation

In previous studies, enzymes have typically been modeled as undergoing first order degradation with a universal constant [33, 34]. However, rates can vary greatly among different proteins [35, 36], and some evidence suggests that H_2_O_2_ detoxification enzymes, such as catalase and alkyl hydroperoxidase, can be poisoned by their own substrate [37–39]. Inactivation of catalase has been described using bimolecular kinetics [40], but it has also been suggested that poisoning should be of the same general form as the enzyme’s reaction kinetics [37]. The AhpC component of alkyl hydroperoxidase is reduced by AhpF after oxidation by H_2_O_2_. If there is not enough NADH present or AhpC reacts with more H_2_O_2_ before encountering AhpF, the cysteine sulfenic acid formed by the first oxidation can be further oxidized to sulfinic or sulfonic acid, rendering it inactive [38, 97]. Whether AhpC inactivation is significant at the concentrations used in this work was uncertain [39]. We therefore allowed it to be degraded in a first order or bimolecular manner. Due to the indeterminacy of how these enzymes are degraded, we included all of these possible degradation scenarios. Rate equations can be found in S2 Table. We note that since AHP requires a co-substrate, NADH, and that we assume that NADH is constant (unless otherwise noted), if AHP inactivation were found to be important it could reflect degradation of AHP or reduced availability of NADH (Reaction 71).

Parameters or bounds on parameters for enzyme degradation/deactivation rate equations varied with the method of degradation. For constant degradation with a constrained constant, we used the "general" protein degradation rate reported by Kotte and colleagues [34]. When optimized, the constant degradation rate had bounds set by the longest [35] and shortest [36] protein half-life we found in literature. For both bimolecular and the more complex inactivation, bounds were based loosely on rate constants found for *Aspergillus niger* and bovine catalase [37, 40]. Based on the gross difference between the organisms tested in those studies (fungus and mammal) and our own (bacteria), as well as the orders of magnitude difference between the *Aspergillus niger* and bovine catalase rates in both the bimolecular and more complex kinetics studies, these parameters were constrained to two orders of magnitude lower than the lowest reported value, and two orders of magnitude higher than the highest reported value.

### Modeling of H_2_O_2_ gradient across the membrane

While H_2_O_2_ rapidly diffuses across bacterial membranes at a rate similar to that of water, there is evidence for and against the existence of a gradient across the membrane [41, 42]. For example, an Ahp−Kat+ strain cocultured with Ahp−Kat− in the presence of a low H_2_O_2_ concentration can outcompete its scavenging deficient neighbors and multiply under the stress, whereas the deficient strain only grows after the catalase proficient strain has cleared the environmental H_2_O_2_ [41]. On the other hand, dilute suspensions of catalase proficient strains are readily killed by high concentrations of H_2_O_2_ similarly to scavenging deficient strains, while high-density catalase proficient strains can not only survive challenge with H_2_O_2_ but also protect deficient neighbors [42]. There have been strides in the ability to measure H_2_O_2_ intracellularly, with the introduction of genetically-encoded indicators (HyPer) [98] and the ability to use Amplex Red intracellularly with expression of a mutated ascorbate peroxidase [99]. However, the dependence of HyPer fluorescence on reductase activity, the impact of HyPer on cellular scavenging capacity [100], and the difficulties associated with converting measurements from either method to absolute H_2_O_2_ concentrations led us to use measurements of external H_2_O_2_ and statistical metrics (AIC) to assess the suitability of modeling the system with or without a gradient. In one set of models, the intracellular and extracellular H_2_O_2_ concentrations were equal. In the other set, we allowed for a gradient by modeling transport across the membrane as a convective mass transport process, with the effective mass transport coefficient being an additional parameter for optimization. The lower bound on the effective mass transfer coefficient was set as the permeability coefficient of H_2_O_2_ across *E*. *coli* cell membranes in unstirred culture [41], adjusted for cell area and the cell density for our system. The upper bound was set two orders of magnitude higher than the permeability, to account for increased mass transfer in our chaotic shake flask system.

### Spontaneous degradation of H_2_O_2_

The rate of spontaneous degradation of H_2_O_2_ into H_2_O and O_2_ was determined using the MATLAB function *lsqcurvefit* after monitoring H_2_O_2_ concentration over time in cell-free controls for each media condition (M9 10 mM glucose with and without CAM and M9 lacking glucose). Samples were collected at time 0, 20 min, 40 min, and 60 min for 10 and 25 μM H_2_O_2_-containing flasks; time 0, 1 h, 2 h, and 3 h for 100 μM H_2_O_2_-containing flasks; and 0 h, 1 h, 2 h, 3 h, and 4 h for 400 μM H_2_O_2_-containing flasks. All control data for each media condition was fit simultaneously (*e*.*g*., 10, 25, 100, and 400 μM H_2_O_2_ in M9 10 mM glucose) while accounting for experimental error by weighting points in the sum squared of residuals (SSR) calculation by the inverse of the variance for that data point [43, 101–103]. The optimized rate constants were 0.0324 h^-1^ for M9 10 mM glucose, 0.0331 h^-1^ for M9 10 mM glucose with 100 μg/mL CAM, and 0 h^-1^ for M9 lacking glucose. The spontaneous degradation rate was assumed to be equivalent in the intracellular and media compartments.

### Other reactions

Other reactive oxygen species in the reaction network include O_2_^-^• and •OH. The model includes endogenous production of O_2_^-^• (Reaction 4) and its dismutation to H_2_O_2_ (Reactions 3, 74–75), as well as its reactions with other molecules (Reactions 1, 25, 26, 55) and production by other reactions in the network (Reaction 49). Additionally, the Fenton reaction produces •OH (Reaction 24), which can react with amino acids (Reactions 5–23) and other compounds in the network (Reactions 1, 27, 36, 37, 48). The rate constant for the Fenton reaction were variable in literature, so bounds were set as the slowest and fastest reported rates [21].

Other reactions include glutathione oxidation, reduction, and reaction with other molecules (Reactions 29–32, 42–55), and oxidation of methionine (Reaction 2) and pyruvate (Reaction 57) by H_2_O_2_. All spontaneous reactions, rate equations, and rate constant can be found in S2 Table [21, 34–37, 39–41, 81, 85, 88, 91, 92, 104–111]. Enzymatic rate equations and rate constants can be found in S3 Table [38, 91, 92, 94, 111–117], and include methionine sulfoxide reductase (Reaction 64), thiol peroxidases (Reaction 65–66), thioredoxin reductase (Reaction 72), and glutathione reductase (Reaction 73).

### Parameter optimization

Parameters were optimized by minimizing the SSR using the built-in MATLAB function *lsqcurvefit*. Because experimental error varied for each time point, we weighted each data point’s contribution to the SSR by the inverse of the variance of that point [43, 101–103]. The initial concentration of H_2_O_2_ in the model was changed to match the experimental data before optimizing. Due to the nonlinear nature of the optimization, each model structure was initialized with random parameter sets within the defined bounds a total of 1,000 times. The progression of minimum SSR found by each of these iterations is shown in S14 Fig. A plateau suggests that additional iterations would not lead to substantial improvement in the fit. The slowest converging optimization reached a plateau after 639 optimizations.

The number of parameters optimized varied according to model structure, as presented in Table 1. Parameters related to HPI and AHP expression (4 parameters total) were optimized in all structures. The Fenton reaction rate constant varied in literature (1 parameter), and intracellular Fe^2+^ and Fe^3+^ concentrations (2 parameters) were unresolved. Additionally, initial concentrations of the major detoxifying enzymes (3 parameters) were unknown and can vary with growth environment and stage of cell growth. Beyond these 10 parameters, all structures that did not have constant enzyme degradation with a universal degradation constant added 3 parameters, and including an H_2_O_2_ gradient added 1 parameter (a convective mass transfer coefficient).

### Model ranking and selection

The introduction of additional parameters, such as enzyme degradation rate constants and mass transport coefficients, has the potential to improve fit solely by increasing the flexibility of the model. To account for the utility of additional parameters, we ranked models based on their evidence ratios (ER), or likeliness relative to the most likely model in the set. For each model, we calculated its Akaike Information Criterion corrected for small sample size (AIC_c_) [43]:

$$AIC_{c}=n\cdot \text{ln}(\frac{SSR}{n})+2K+\frac{2K(K+1)}{n-K-1}$$ (7)

where *n* represents the sample size and *K* is the number of estimable parameters. Here, *n* is the number of data points used in the fitting procedure, and *K* is the number of model parameters plus 1 because regression estimates SSR and parameter values [43]. We account for unequal variances within the data by using weighted least squares, where each point is weighted by the inverse of its variance.

The weight of evidence for a given model in a set of *M* models is given by the following [43]:

$$w_{i}=\frac{e^{-\Delta_{i}/2}}{\sum_{i=1}^{M}e^{-\Delta_{i}/2}}$$ (8)

where *Δ*_*i*_ = *AIC*_*i*_-min(*AIC*). With this, an ER can be calculated, which represents the relative likelihood of a model compared to the best model in the set [27]:

$$ER_{i}=\frac{w_{best}}{w_{i}}$$ (9)

A larger ER indicates a more unlikely model. In this work, models with an ER greater than 10 were discarded, a cutoff that has been used previously to discard models during model selection [29].

### Generation of the ensemble

To account for parametric uncertainty when making predictions, we generated an ensemble of parameter sets that all predicted the data within an ER≤10. We initially attempted to use the software HYPERSPACE [26], which is a three-step process that provides a uniform sampling of the viable parameter space. However, the calculation of our cost function was computationally expensive, which lengthened the time required per iteration, and the method had not converged within 100,000 iterations. Therefore, we utilized the pre-existing Markov chain Monte Carlo function within the HYPERSPACE software, but started it from all 40 viable parameter sets that met our design criteria. We allowed approximately 200 random steps away from each viable point, and randomly selected 100 of those parameters sets with an ER≤10 from each random walk. This process generated 4,000 parameter sets that had an ER≤10.

### Identification of the minimal H_2_O_2_ model

We identified the minimal model that was able to capture our data (ER≤10) using a previously developed two tiered approach [33]. In the first tier, reactions were removed from the best model in a random order and the ER was calculated. If the deletion of a reaction increased the ER above its threshold of 10, it was returned to the model and the process continued through the remaining reactions. This random deletion process was repeated 100 times. In the second tier, parameters were re-optimized after the deletion of each of the remaining reactions. If the optimization produced a model that returned below an ER of 10, the parameters were changed and the process continued.

## Supporting Information

S1 Fig CFUs/mL during clearance assays. **A-D.** CFUs/mL during wild-type clearance of 10 (A), 25 (B), 100 (C), and 400 (D) μM H_2_O_2_ in M9 10 mM glucose media. **E-H.** CFUs/mL during Δ*katE* Δ*katG* clearance of 10 (E), 25 (F), 100 (G), and 400 (H) μM H_2_O_2_ in M9 10 mM glucose media. **I-L.** CFUs/mL during Δ*katE* clearance of 10 (I), 25 (J), 100 (K), and 400 (L) μM H_2_O_2_ in M9 10 mM glucose media. **M-P.** CFUs/mL during Δ*katG* clearance of 10 (M), 25 (N), 100 (O), and 400 (P) μM H_2_O_2_ in M9 10 mM glucose media. **Q-T.** CFUs/mL during wild-type clearance of 10 (Q), 25 (R), 100 (S), and 400 (T) μM H_2_O_2_ in M9 media lacking glucose. **U-X.** CFUs/mL during wild-type clearance of 10 (U), 25 (V), 100 (W), and 400 (X) μM H_2_O_2_ in M9 10 mM glucose media with 100 μg/mL CAM. **Y.** CFUs/mL during *ΔkatE* Δ*katG* clearance of 10 μM H_2_O_2_ in M9 media lacking glucose. **Z.** CFUs/mL during Δ*katE* Δ*katG* clearance of 10 μM H_2_O_2_ in M9 10 mM glucose media with 100 μg/mL CAM. **AA.** CFUs/mL during Δ*ahpCF* Δ*katE* Δ*katG* clearance of 10 μM H_2_O_2_ in M9 10 mM glucose media. Experiments were performed with three biological replicates. Error bars show the standard error of the mean. Asterisks indicate significant (p<0.05) CFU loss from the initial value based on a two-tailed t-test with unequal variance performed on log-transformed values.
(TIF)

S2 Fig Reaction flux through AHP and HPI+HPII. Reaction flux through the two major detoxification systems AHP vs. HPI+HPII are shown as a function of time. **A-D**. Reaction fluxes for the 35 acceptable models after fitting on wild-type data (Fig 3). **E-H.** Reaction fluxes for the 965 acceptable models after fitting simultaneously on wild-type and Δ*katE* Δ*katG* data (Fig 4). **I-L.** Reaction fluxes for the 40 acceptable models after fitting on wild-type, Δ*katE* Δ*katG*, Δ*katE*, and Δ*katG* data (Fig 5). Each line represents the prediction from a single model.
(TIF)

S3 Fig Reaction flux through HPI and HPII. Reaction flux through the two catalases HPI and HPII are shown as a function of time. **A-D.** Reaction fluxes for the 965 acceptable models after fitting simultaneously on wild-type and Δ*katE* Δ*katG* data (Fig 4). **E-H.** Reaction fluxes for the 40 acceptable models after fitting on wild-type, Δ*katE* Δ*katG*, Δ*katE*, and Δ*katG* data (Fig 5). Each line represents the prediction from a single model.
(TIF)

S4 Fig Prediction for H_2_O_2_ clearance by Δ*katE* and Δ*katG*. Predicted clearance of 10 (A), 25 (B), 100 (C), and 400 (D) μM H_2_O_2_ by Δ*katE*, and 10 (E), 25 (F), 100 (G), and 400 (H) μM H_2_O_2_ by Δ*katG* in M9 10 mM glucose media. Each line represents the prediction from one of the 965 acceptable models trained on wild-type and Δ*katE* Δ*katG* H_2_O_2_ clearance in M9 10 mM glucose media (Fig 4). Wide distributions on clearance dynamics suggest that these single mutants could be used to discriminate between models.
(TIF)

S5 Fig Ensemble consistency. To ensure that none of the models in our ensemble violated the design criteria, we checked the consistency of predictions for H_2_O_2_ distribution across the detoxification pathways for the 4,000 model set. **A-D.** Prediction for the amount of H_2_O_2_ cleared by the two major detoxification pathways AHP (orange) and combined catalase activity (black) after boluses of 10 (**A**), 25 (**B**), 100 (**C**), and 400 (**D**) μM H_2_O_2_. Each line represents the prediction from a single model. **I-L.** Prediction for the amount of H_2_O_2_ cleared by the individual catalases HPI (pink) and HPII (green) after boluses of 10 (**E**), 25 (**F**), 100 (**G**), and 400 (**H**) μM H_2_O_2_. Each line represents the prediction from a single model.
(TIF)

S6 Fig Parameter sensitivity analysis. Beginning from the best parameter set in our ensemble, parameters were varied between their bounds. Parameters that increased the ER to beyond our threshold of 10 are shown in the figure. The Fenton reaction rate constant and Fe^2+^ and Fe^3+^ initial concentrations did not substantially affect the ER.
(TIF)

S7 Fig [NAD+] and [NADH] dependence on glucose availability. Exponentially growing cells were transferred to fresh M9 10 mM glucose or M9 lacking carbon. Time 0- points were measured before resuspension in fresh media. Data represents the average of four biological replicates, and error bars show the standard error of the mean. Cells have a significantly lower NADH level after 60 minutes in carbon-free media (p = 0.035), as determined by a two-tailed t-test with unequal variance. A higher cell density (OD_600_ = 0.2) than that used in the H_2_O_2_ clearance assays was necessary to exceed the limit of detection of the kit (BioAssay Systems EnzyChrom^TM^ NAD/NADH Assay Kit).
(TIF)

S8 Fig Dependence of respiration on glucose availability. Exponentially growing cells were washed, resuspended in media with or without glucose, and used to inoculate M9 media +/- glucose to an OD_600_ of 0.1. A higher density than that used for H_2_O_2_ clearance assays was necessary to observe a measureable drop in O_2_. Cells were allowed to consume oxygen for ten minutes before being treated with 5 mM KCN to inhibit respiration. The solid lines show the average of three biological replicates, and windows represent the standard error of the mean. We found that cultures in M9 media with 10 mM glucose efficiently consumed oxygen via respiration, whereas glucose deprived cultures consumed very little.
(TIF)

S9 Fig AHP activity is removed in glucose-deprived cultures treated with 10 μM H_2_O_2_. To explore whether omitting glucose from the media effectively eliminated AHP activity in a regime where it dominates (10 μM H_2_O_2_), we compared H_2_O_2_ clearance in Δ*katE* Δ*katG* in M9 minimal media with glucose (blue), without glucose (orange), and with glucose and 100 μg/mL CAM (black) to Δ*katE* Δ*katG* Δ*ahpCF* (red) and cell-free controls (green). Removal of the two other major detoxification systems leaves AHP, which requires one NADH for every reaction cycle, as the only major detoxification system. When glucose is omitted from the media, H_2_O_2_ clearance is eliminated, as evidenced by Δ*katE* Δ*katG* in M9 minimal media without glucose never differing significantly from Δ*ahpCF* Δ*katE* Δ*katG* or a cell-free control based on a two-tailed t-test with unequal variance. These results were not due solely to inhibition of translation, another effect of glucose starvation (S11 Fig), based on clearance abilities of CAM-treated, Δ*katE* Δ*katG* cultures.
(TIF)

S10 Fig Predicted NADH concentration when its levels are not maintained. Under conditions of glucose deprivation, AHP drains NADH in less than a second after exposure to 10 (A), 25 (B), 100 (C), and 400 (D) μM H_2_O_2_. Windows show the maximum and minimum predictions of the 4,000 models in the ensemble. The solid line indicates the prediction made by the most likely model. Note that the x-axis here is in seconds, not hours. We note that in all other simulations, the NADH concentration was held constant (S1 Table).
(TIF)

S11 Fig Synthesis of GFP under control of *katE*, *katG*, and *ahpC* promoters. Wild-type cells transformed with pUA66 P_*katE*_-*gfp* (A-D), pUA66 P_*katG*_-*gfp* (F-I), or pUA66 P_*ahpC*_-*gfp* (J-M) were exposed to H_2_O_2_ in M9 10 mM glucose (blue), M9 lacking glucose (red), or M9 10 mM glucose + 100 μg/mL CAM (green) media. We confirmed that the lack of expression of GFP from pUA66 P_*katE*_-*gfp* after exposure to H_2_O_2_ was not due to a defect in the vector by including a 16 h overnight control in M9 10 mM glucose. As expected, *katE* expression increases in stationary phase as determined by an increase in fluorescence after 16 h (0 μM H_2_O_2_ panel). In all cases, an empty vector control (black) was included to account for auto-fluorescence. Two biological replicates were analyzed on different days for each experiment. A representative replicate is shown here. Solid lines indicate time 0- distributions. For the P_*katE*_-*gfp* control, the time 0- line in the 0 μM H_2_O_2_ panel represents inoculation after an 8 h growth period to mid-exponential phase. Dashed lines show distributions after ~90% of the H_2_O_2_ has been cleared by wild-type in M9 10 mM glucose (see Methods), or 16 h for the P_*katE*_-*gfp* control (0 μM H_2_O_2_ panel).
(TIF)

S12 Fig H_2_O_2_ measurement after centrifuging and/or sterile filtering to remove cells. For the 30 min point in the 400 μM H_2_O_2_ clearance assay, three aliquots of sample were removed. One was centrifuged for 3 min at 15,000 rpm and the supernatant was removed identically to our protocol, one was left on the bench during the 3 min spin and then sterile filtered with an 0.22 μM syringe filter, and one was centrifuged and then sterile filtered. The experiment was performed in triplicate. Error bars show the standard error of the mean. Measurements from centrifuging alone were not significantly different from filtering (p = 0.45) or centrifuging + filtering (p = 0.40) based on a two-sample t-test with unequal variance.
(TIF)

S13 Fig Clearance assays without exogenous catalase. To determine whether exogenous catalase in the overnight or flask growth prior to the assay affected H_2_O_2_ clearance profiles for wild-type *E*. *coli*, we performed clearance assays in M9 media with 10 mM glucose after omitting the catalase in all pre-processing steps. **A-D.** H_2_O_2_ concentration did not differ significantly at any time point. **E-H.** CFU loss was not observed under either condition.
(TIF)

S14 Fig Minimum sum of squared residuals (SSR) with increasing optimizations performed. Each parameter set optimization was randomly initialized 1,000 times. Presented here is the minimum SSR that had been found at each of the 1,000 iterations (model index) for the following optimizations performed: model structure 1 (A), 2 (B), 3 (C), 4 (D), 5 (E), 6 (F), 7 (G), 8 (H), 9 (I), 10 (J) trained on wild-type data; model structure 2 (K) and 3 (L) trained on wild-type and Δ*katE* Δ*katG* data; model structure 3 (M) on wild-type, Δ*katE* Δ*katG*, Δ*katE*, and Δ*katG* data. This data suggested that more than 1,000 initializations would provide very little return for additional computational time invested.
(TIF)

S1 Table Model species. All metabolites and enzymes are listed with their initial concentrations, references, and relevant notes. Bounds are listed for uncertain parameters.
(XLSX)

S2 Table Spontaneous reactions. All spontaneous reactions are provided with their rate constants, references, and relevant notes. Bounds are listed for uncertain parameters.
(DOCX)

S3 Table Enzymatic reactions. All enzymatic reactions are provided with their rate equations, constants, and references. Bounds are listed for uncertain parameters, which are indicated by an asterisk.
(DOCX)

S4 Table Primer sequences. (DOCX)
