# Supplementary figures and images for: A Kinetic Platform to Determine the Fate of Hydrogen Peroxide in Escherichia coli
Source: PLoS Comput Biol. 2015 Nov 6;11(11):e1004562. doi: 10.1371/journal.pcbi.1004562 (PMC4636272; doi:10.1371/journal.pcbi.1004562)

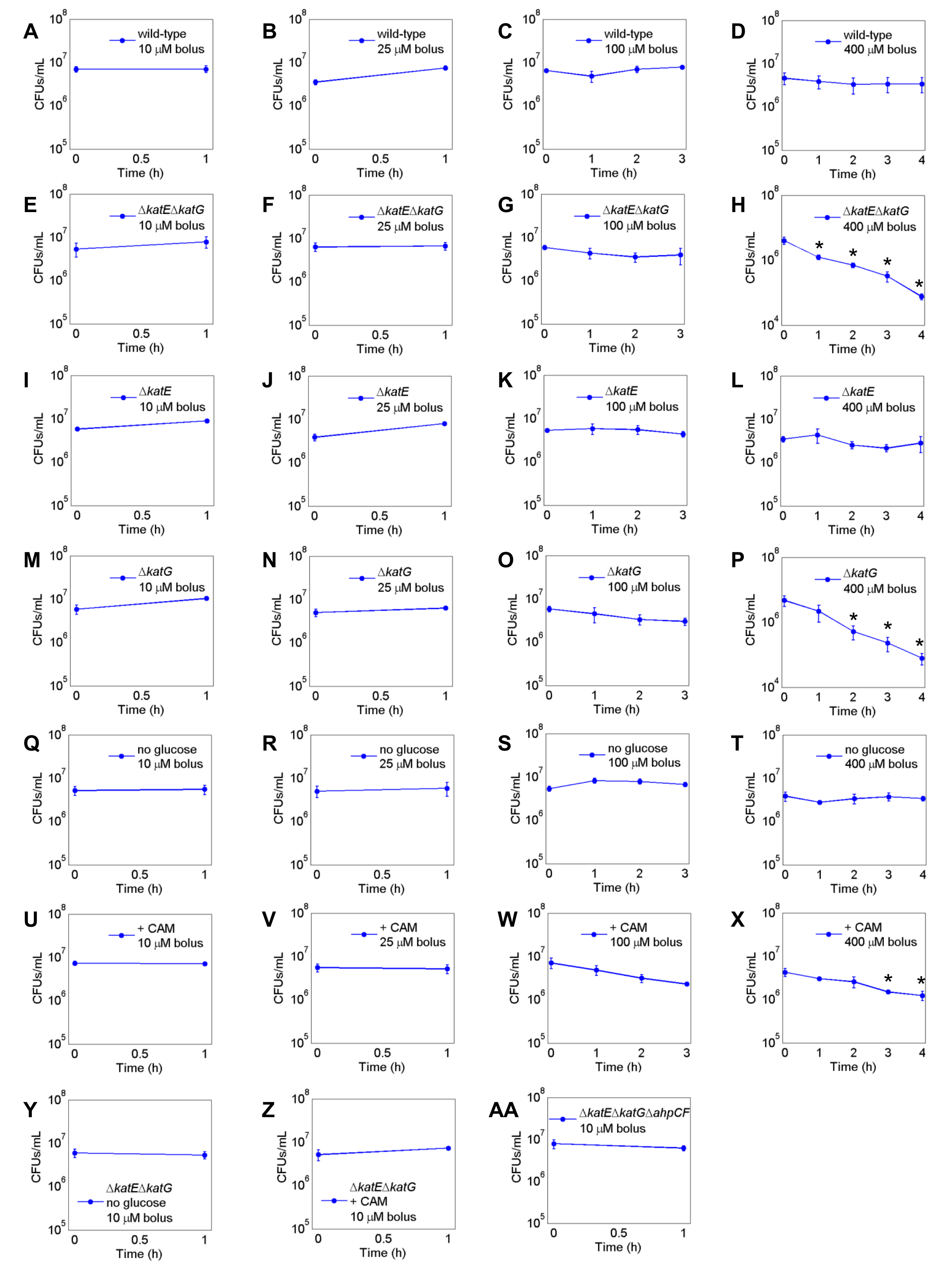

Supplement: S1 Fig — A-D. CFUs/mL during wild-type clearance of 10 (A), 25 (B), 100 (C), and 400 (D) μM H2O2 in M9 10 mM glucose media. E-H. CFUs/mL during ΔkatE ΔkatG clearance of 10 (E), 25 (F), 100 (G), and 400 (H) μM H2O2 in M9 10 mM glucose media. I-L. CFUs/mL during ΔkatE clearance of 10 (I), 25 (J), 100 (K), and 400 (L) μM H2O2 in M9 10 mM glucose media. M-P. CFUs/mL during ΔkatG clearance of 10 (M), 25 (N), 100 (O), and 400 (P) μM H2O2 in M9 10 mM glucose media. Q-T. CFUs/mL during wild-type clearance of 10 (Q), 25 (R), 100 (S), and 400 (T) μM H2O2 in M9 media lacking glucose. U-X. CFUs/mL during wild-type clearance of 10 (U), 25 (V), 100 (W), and 400 (X) μM H2O2 in M9 10 mM glucose media with 100 μg/mL CAM. Y. CFUs/mL during ΔkatE ΔkatG clearance of 10 μM H2O2 in M9 media lacking glucose. Z. CFUs/mL during ΔkatE ΔkatG clearance of 10 μM H2O2 in M9 10 mM glucose media with 100 μg/mL CAM. AA. CFUs/mL during ΔahpCF ΔkatE ΔkatG clearance of 10 μM H2O2 in M9 10 mM glucose media. Experiments were performed with three biological replicates. Error bars show the standard error of the mean. Asterisks indicate significant (p<0.05) CFU loss from the initial value based on a two-tailed t-test with unequal variance performed on log-transformed values. (TIF) [file pcbi.1004562.s001.tif]

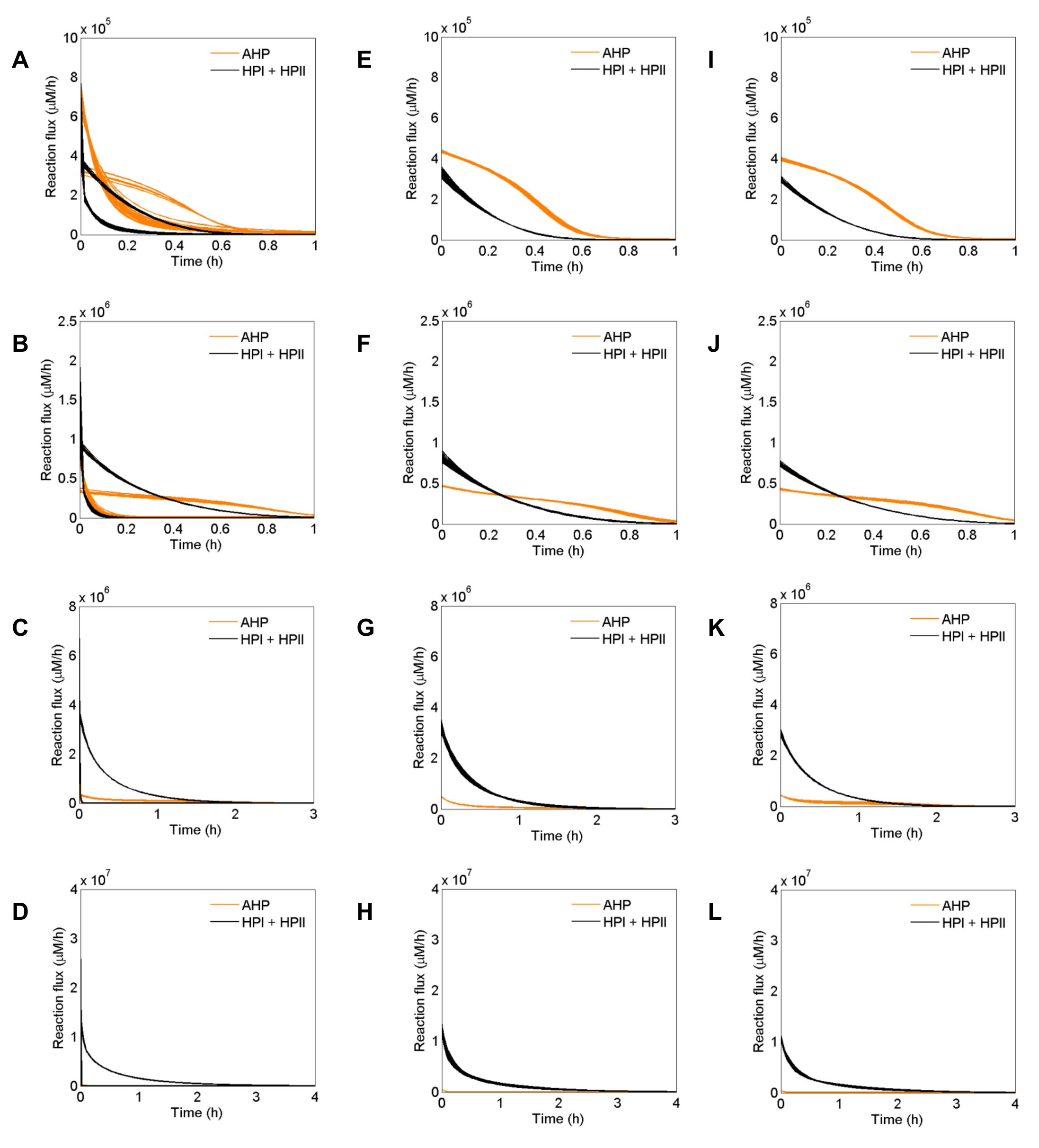

Supplement: S2 Fig — Reaction flux through the two major detoxification systems AHP vs. HPI+HPII are shown as a function of time. A-D. Reaction fluxes for the 35 acceptable models after fitting on wild-type data (Fig 3). E-H. Reaction fluxes for the 965 acceptable models after fitting simultaneously on wild-type and ΔkatE ΔkatG data (Fig 4). I-L. Reaction fluxes for the 40 acceptable models after fitting on wild-type, ΔkatE ΔkatG, ΔkatE, and ΔkatG data (Fig 5). Each line represents the prediction from a single model. (TIF) [file pcbi.1004562.s002.tif]

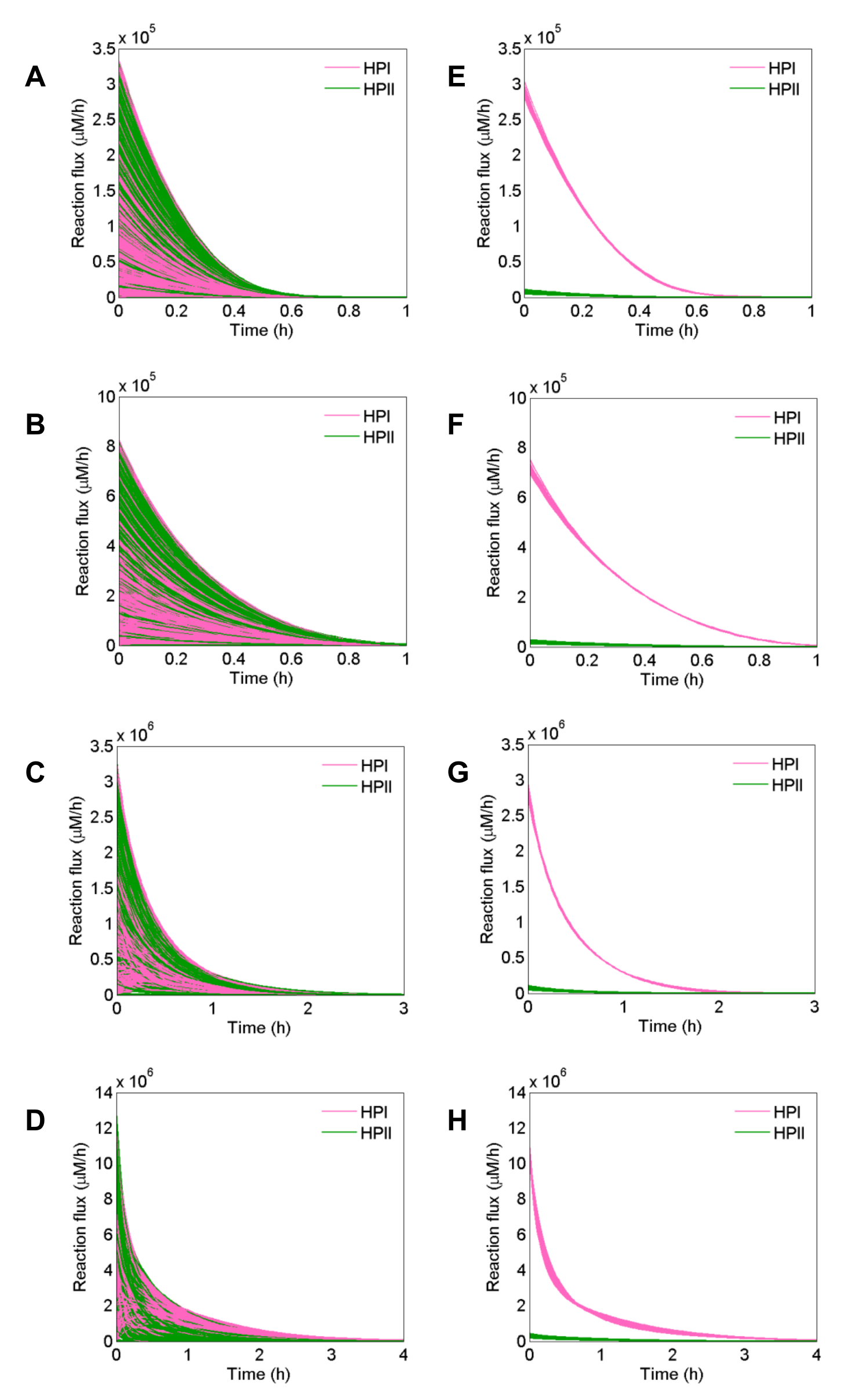

Supplement: S3 Fig — Reaction flux through the two catalases HPI and HPII are shown as a function of time. A-D. Reaction fluxes for the 965 acceptable models after fitting simultaneously on wild-type and ΔkatE ΔkatG data (Fig 4). E-H. Reaction fluxes for the 40 acceptable models after fitting on wild-type, ΔkatE ΔkatG, ΔkatE, and ΔkatG data (Fig 5). Each line represents the prediction from a single model. (TIF) [file pcbi.1004562.s003.tif]

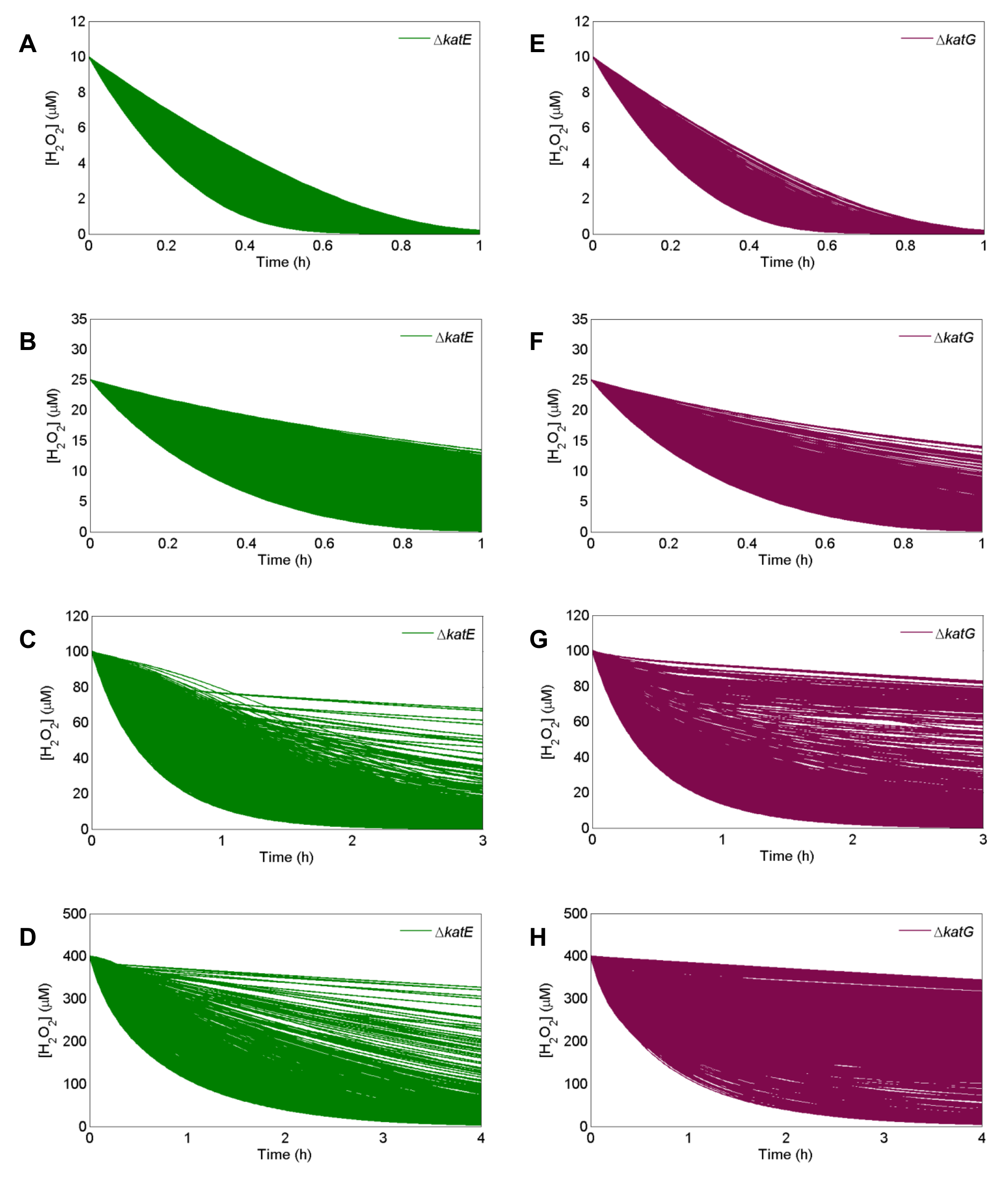

Supplement: S4 Fig — Predicted clearance of 10 (A), 25 (B), 100 (C), and 400 (D) μM H2O2 by ΔkatE, and 10 (E), 25 (F), 100 (G), and 400 (H) μM H2O2 by ΔkatG in M9 10 mM glucose media. Each line represents the prediction from one of the 965 acceptable models trained on wild-type and ΔkatE ΔkatG H2O2 clearance in M9 10 mM glucose media (Fig 4). Wide distributions on clearance dynamics suggest that these single mutants could be used to discriminate between models. (TIF) [file pcbi.1004562.s004.tif]

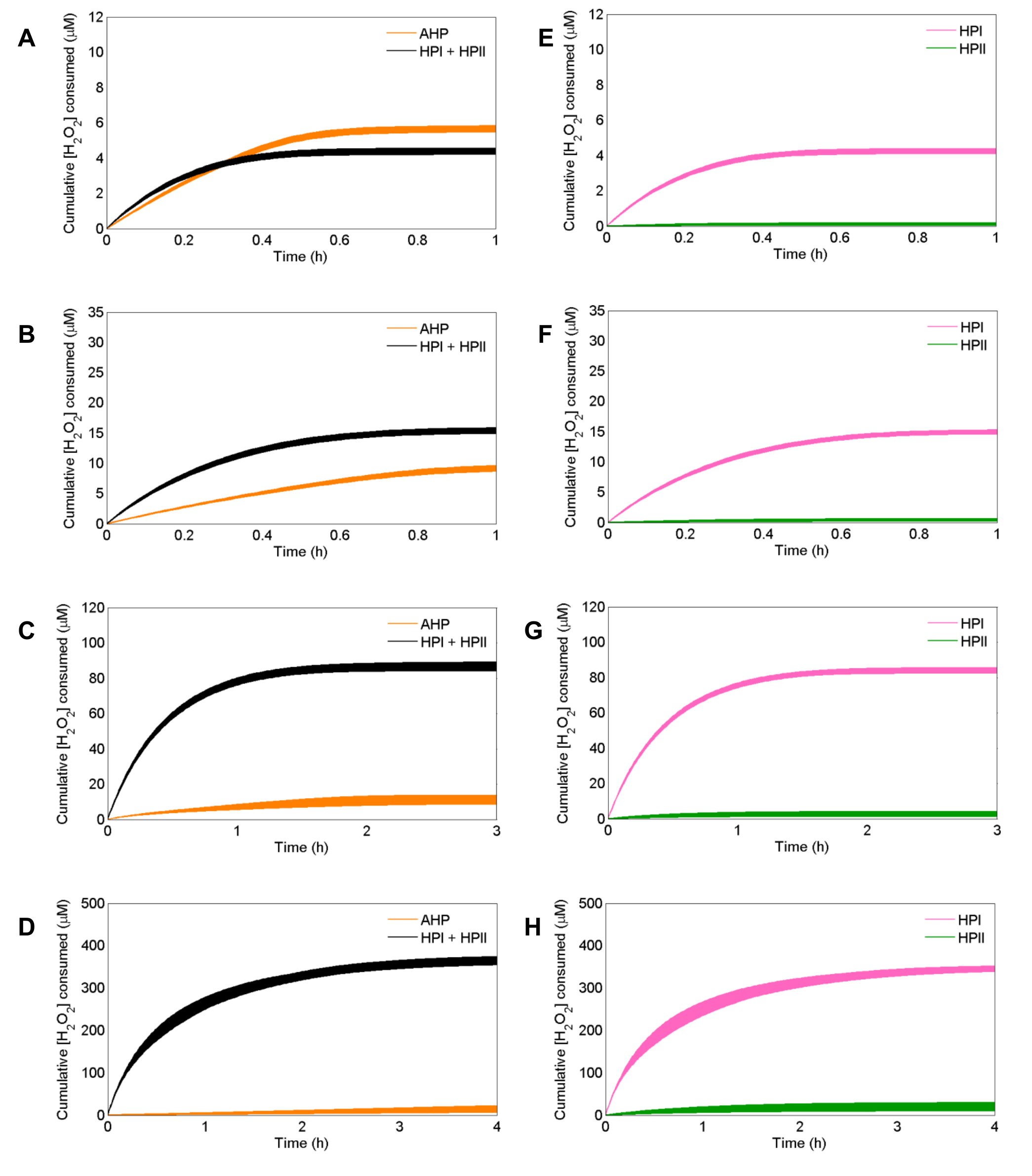

Supplement: S5 Fig — To ensure that none of the models in our ensemble violated the design criteria, we checked the consistency of predictions for H2O2 distribution across the detoxification pathways for the 4,000 model set. A-D. Prediction for the amount of H2O2 cleared by the two major detoxification pathways AHP (orange) and combined catalase activity (black) after boluses of 10 (A), 25 (B), 100 (C), and 400 (D) μM H2O2. Each line represents the prediction from a single model. I-L. Prediction for the amount of H2O2 cleared by the individual catalases HPI (pink) and HPII (green) after boluses of 10 (E), 25 (F), 100 (G), and 400 (H) μM H2O2. Each line represents the prediction from a single model. (TIF) [file pcbi.1004562.s005.tif]

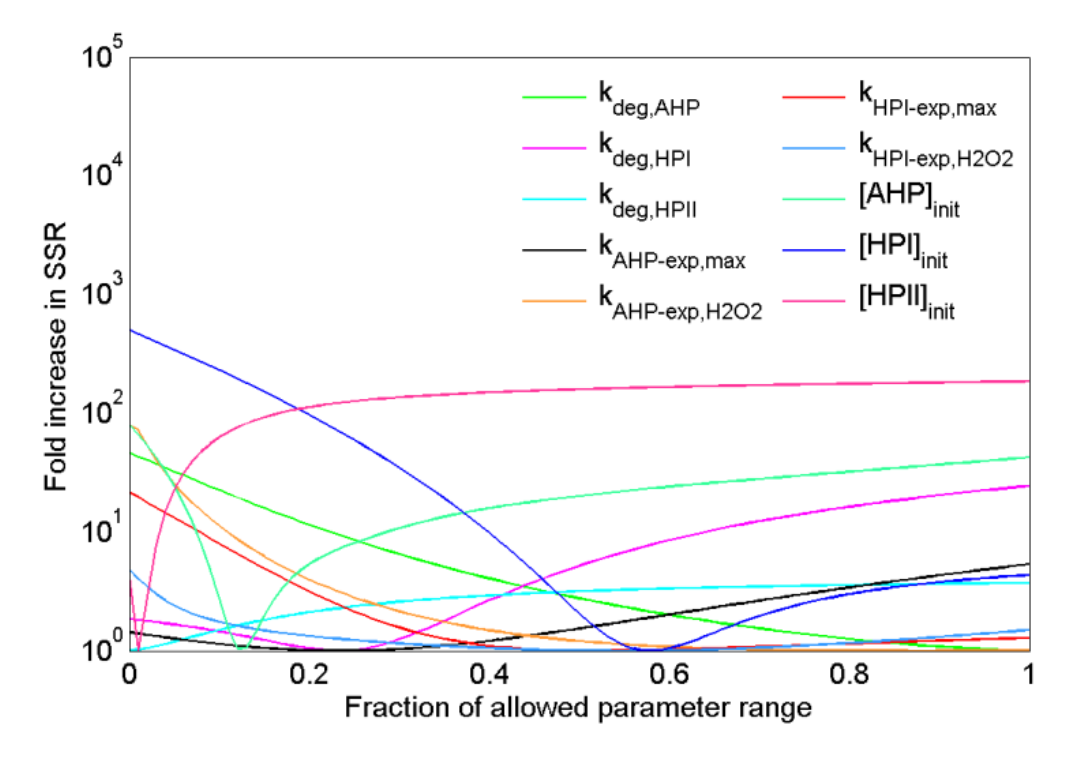

Supplement: S6 Fig — Beginning from the best parameter set in our ensemble, parameters were varied between their bounds. Parameters that increased the ER to beyond our threshold of 10 are shown in the figure. The Fenton reaction rate constant and Fe2+ and Fe3+ initial concentrations did not substantially affect the ER. (TIF) [file pcbi.1004562.s006.tif]

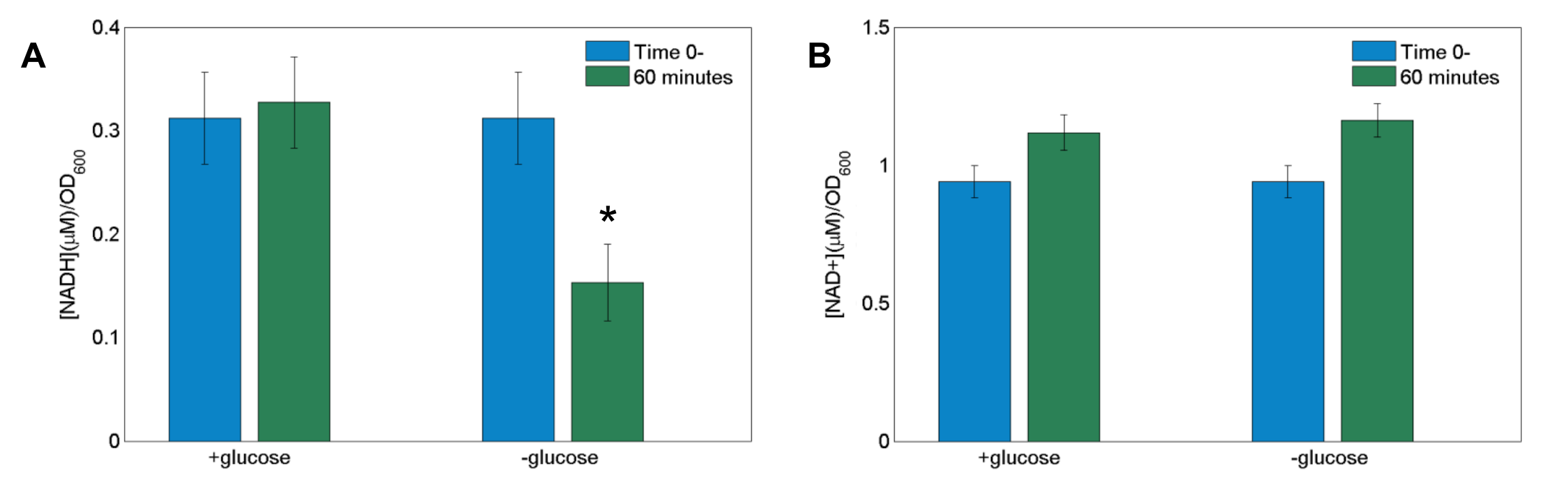

Supplement: S7 Fig — Exponentially growing cells were transferred to fresh M9 10 mM glucose or M9 lacking carbon. Time 0- points were measured before resuspension in fresh media. Data represents the average of four biological replicates, and error bars show the standard error of the mean. Cells have a significantly lower NADH level after 60 minutes in carbon-free media (p = 0.035), as determined by a two-tailed t-test with unequal variance. A higher cell density (OD600 = 0.2) than that used in the H2O2 clearance assays was necessary to exceed the limit of detection of the kit (BioAssay Systems EnzyChromTM NAD/NADH Assay Kit). (TIF) [file pcbi.1004562.s007.tif]

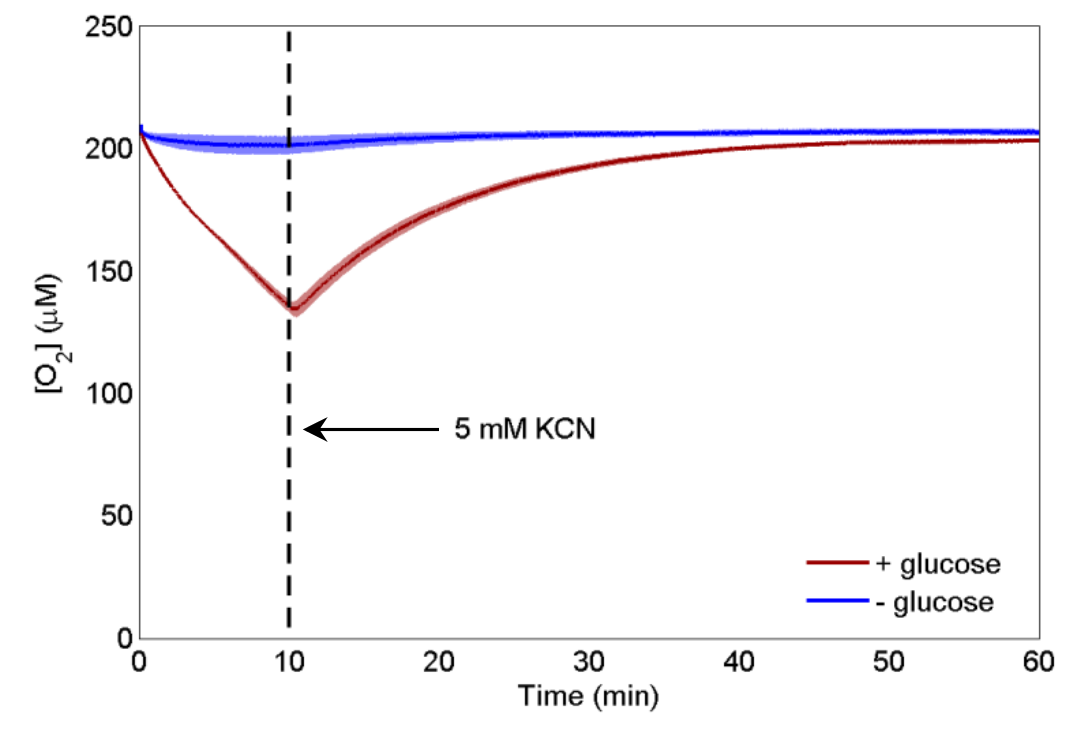

Supplement: S8 Fig — Exponentially growing cells were washed, resuspended in media with or without glucose, and used to inoculate M9 media +/- glucose to an OD600 of 0.1. A higher density than that used for H2O2 clearance assays was necessary to observe a measureable drop in O2. Cells were allowed to consume oxygen for ten minutes before being treated with 5 mM KCN to inhibit respiration. The solid lines show the average of three biological replicates, and windows represent the standard error of the mean. We found that cultures in M9 media with 10 mM glucose efficiently consumed oxygen via respiration, whereas glucose deprived cultures consumed very little. (TIF) [file pcbi.1004562.s008.tif]

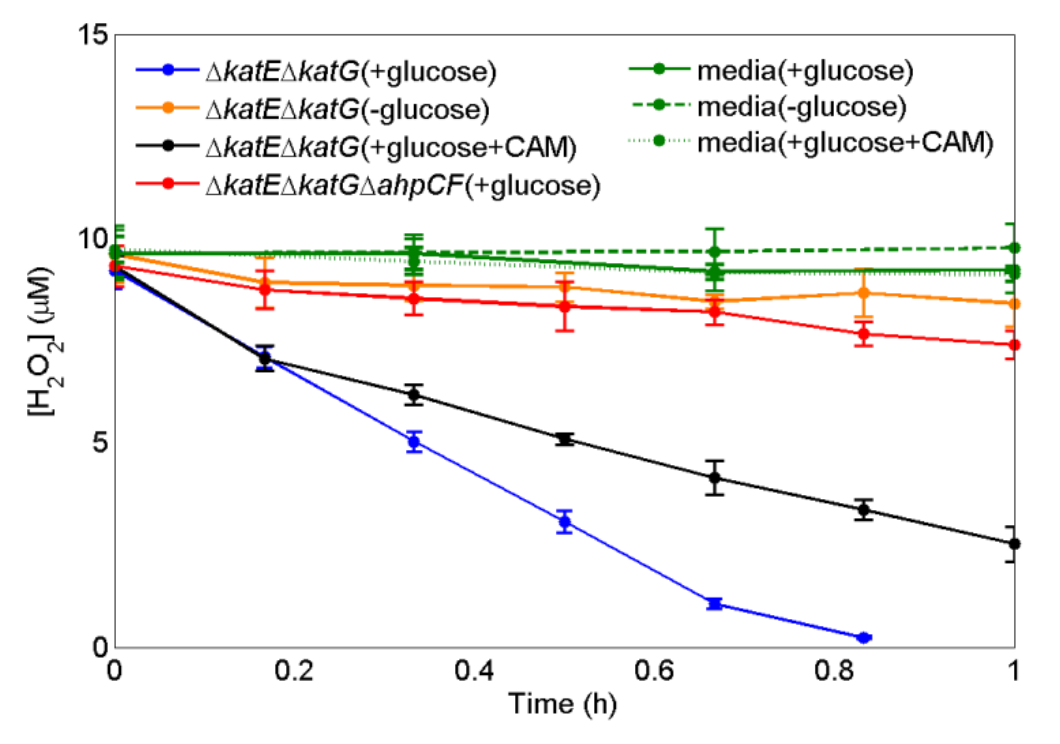

Supplement: S9 Fig — To explore whether omitting glucose from the media effectively eliminated AHP activity in a regime where it dominates (10 μM H2O2), we compared H2O2 clearance in ΔkatE ΔkatG in M9 minimal media with glucose (blue), without glucose (orange), and with glucose and 100 μg/mL CAM (black) to ΔkatE ΔkatG ΔahpCF (red) and cell-free controls (green). Removal of the two other major detoxification systems leaves AHP, which requires one NADH for every reaction cycle, as the only major detoxification system. When glucose is omitted from the media, H2O2 clearance is eliminated, as evidenced by ΔkatE ΔkatG in M9 minimal media without glucose never differing significantly from ΔahpCF ΔkatE ΔkatG or a cell-free control based on a two-tailed t-test with unequal variance. These results were not due solely to inhibition of translation, another effect of glucose starvation (S11 Fig), based on clearance abilities of CAM-treated, ΔkatE ΔkatG cultures. (TIF) [file pcbi.1004562.s009.tif]

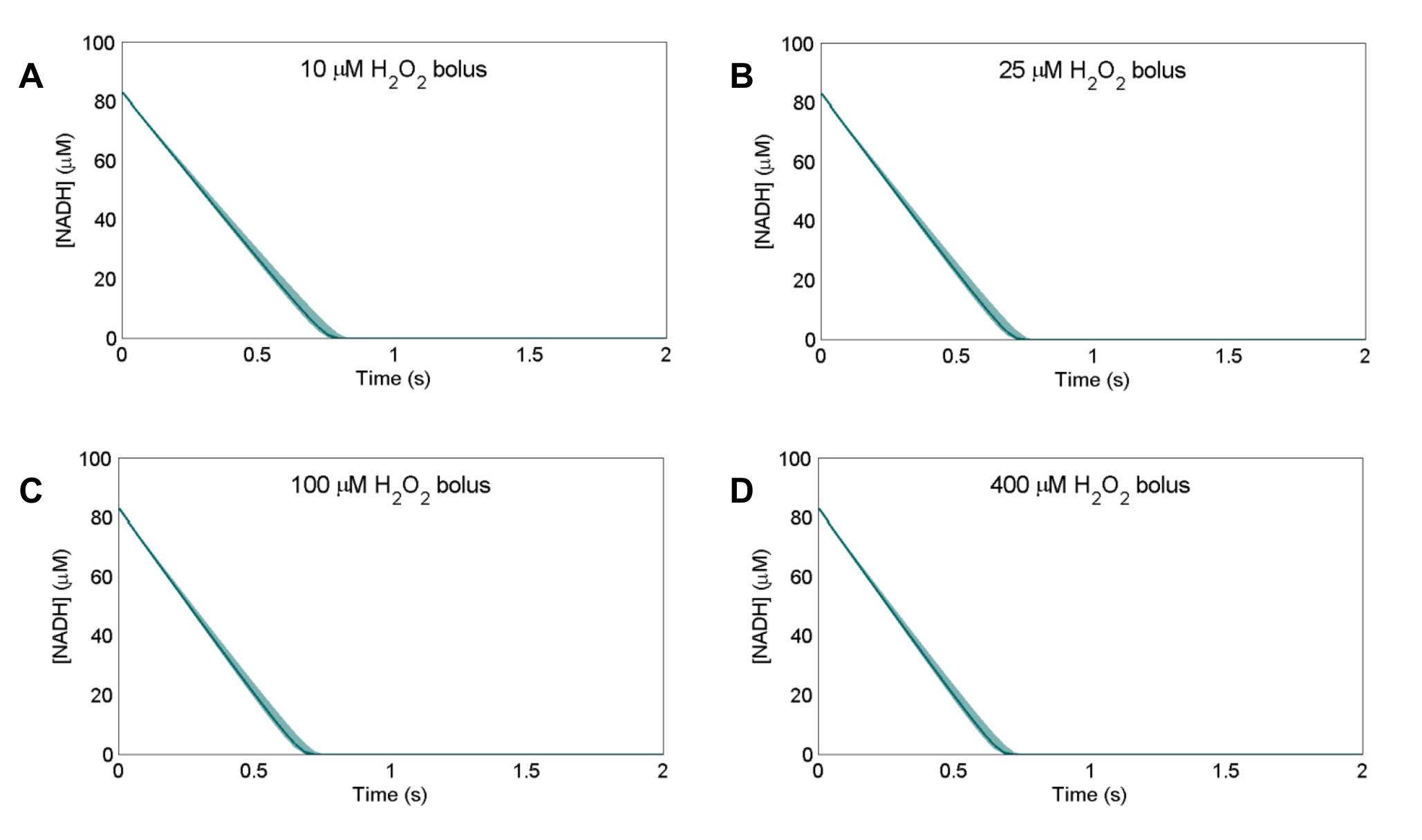

Supplement: S10 Fig — Under conditions of glucose deprivation, AHP drains NADH in less than a second after exposure to 10 (A), 25 (B), 100 (C), and 400 (D) μM H2O2. Windows show the maximum and minimum predictions of the 4,000 models in the ensemble. The solid line indicates the prediction made by the most likely model. Note that the x-axis here is in seconds, not hours. We note that in all other simulations, the NADH concentration was held constant (S1 Table). (TIF) [file pcbi.1004562.s010.tif]

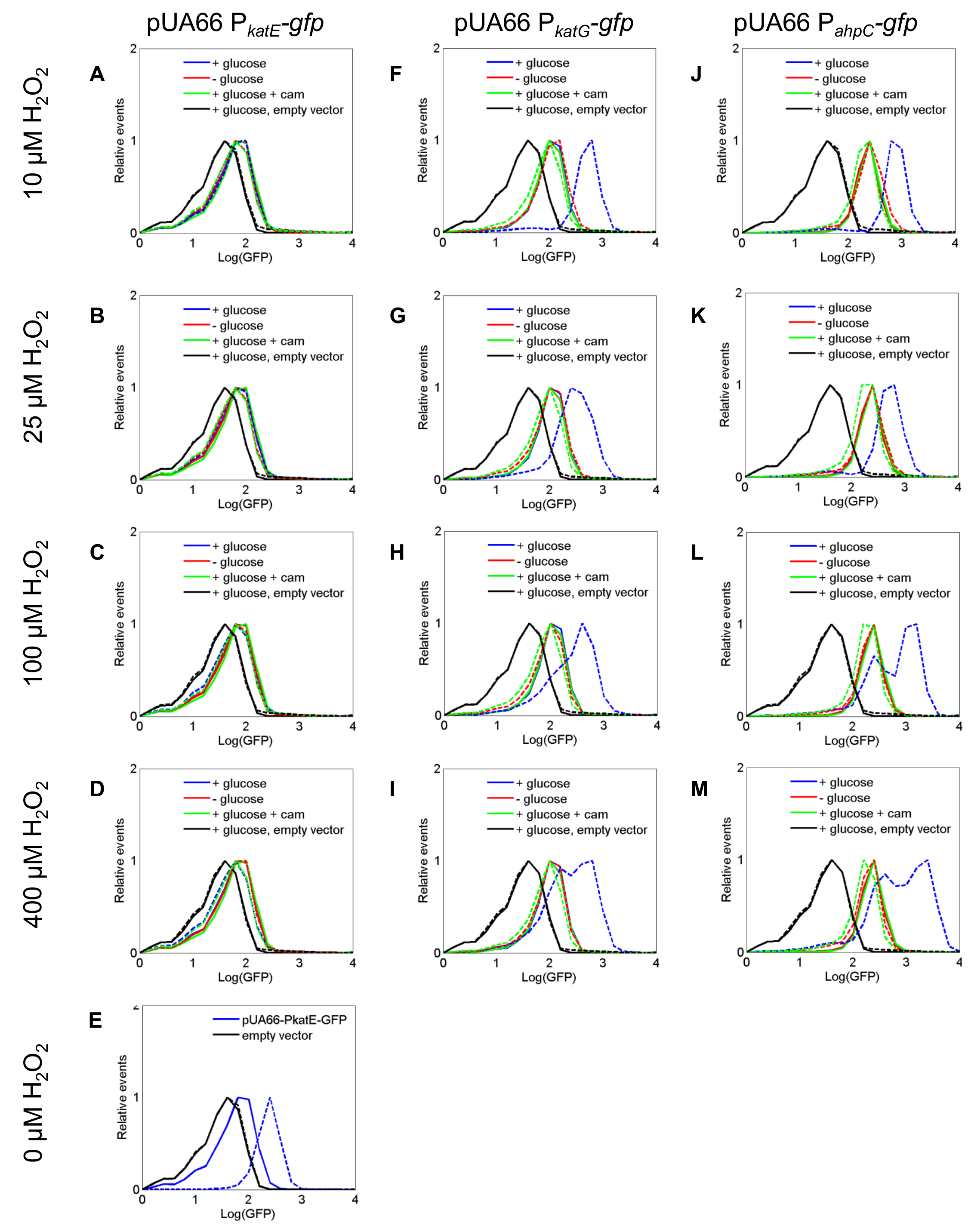

Supplement: S11 Fig — Wild-type cells transformed with pUA66 PkatE-gfp (A-D), pUA66 PkatG-gfp (F-I), or pUA66 PahpC-gfp (J-M) were exposed to H2O2 in M9 10 mM glucose (blue), M9 lacking glucose (red), or M9 10 mM glucose + 100 μg/mL CAM (green) media. We confirmed that the lack of expression of GFP from pUA66 PkatE-gfp after exposure to H2O2 was not due to a defect in the vector by including a 16 h overnight control in M9 10 mM glucose. As expected, katE expression increases in stationary phase as determined by an increase in fluorescence after 16 h (0 μM H2O2 panel). In all cases, an empty vector control (black) was included to account for auto-fluorescence. Two biological replicates were analyzed on different days for each experiment. A representative replicate is shown here. Solid lines indicate time 0- distributions. For the PkatE-gfp control, the time 0- line in the 0 μM H2O2 panel represents inoculation after an 8 h growth period to mid-exponential phase. Dashed lines show distributions after ~90% of the H2O2 has been cleared by wild-type in M9 10 mM glucose (see Methods), or 16 h for the PkatE-gfp control (0 μM H2O2 panel). (TIF) [file pcbi.1004562.s011.tif]

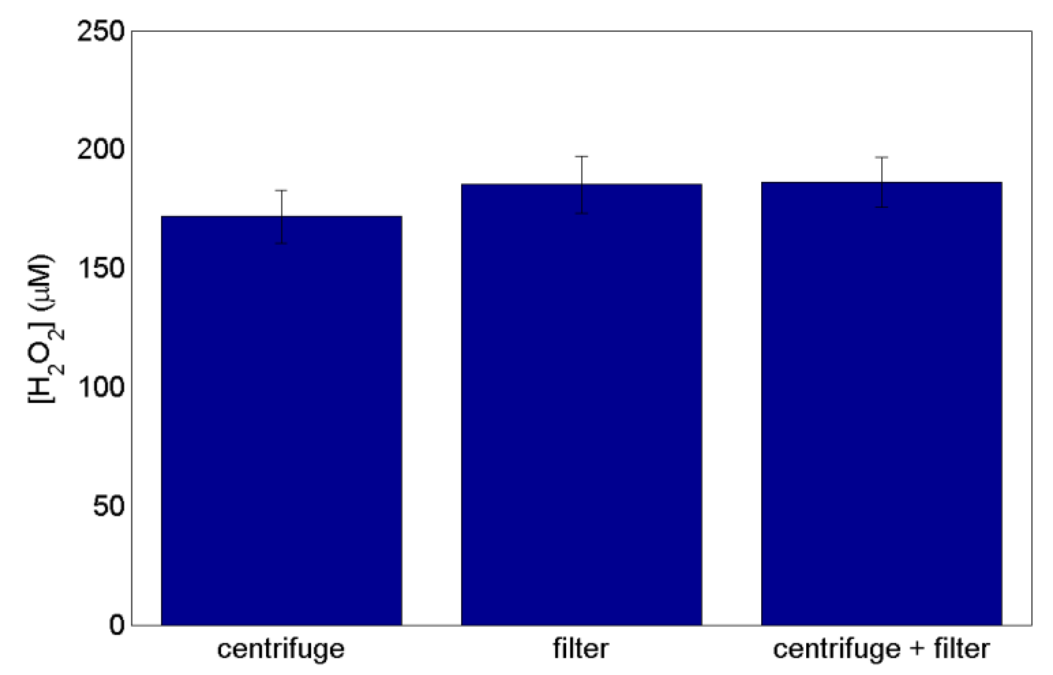

Supplement: S12 Fig — For the 30 min point in the 400 μM H2O2 clearance assay, three aliquots of sample were removed. One was centrifuged for 3 min at 15,000 rpm and the supernatant was removed identically to our protocol, one was left on the bench during the 3 min spin and then sterile filtered with an 0.22 μM syringe filter, and one was centrifuged and then sterile filtered. The experiment was performed in triplicate. Error bars show the standard error of the mean. Measurements from centrifuging alone were not significantly different from filtering (p = 0.45) or centrifuging + filtering (p = 0.40) based on a two-sample t-test with unequal variance. (TIF) [file pcbi.1004562.s012.tif]

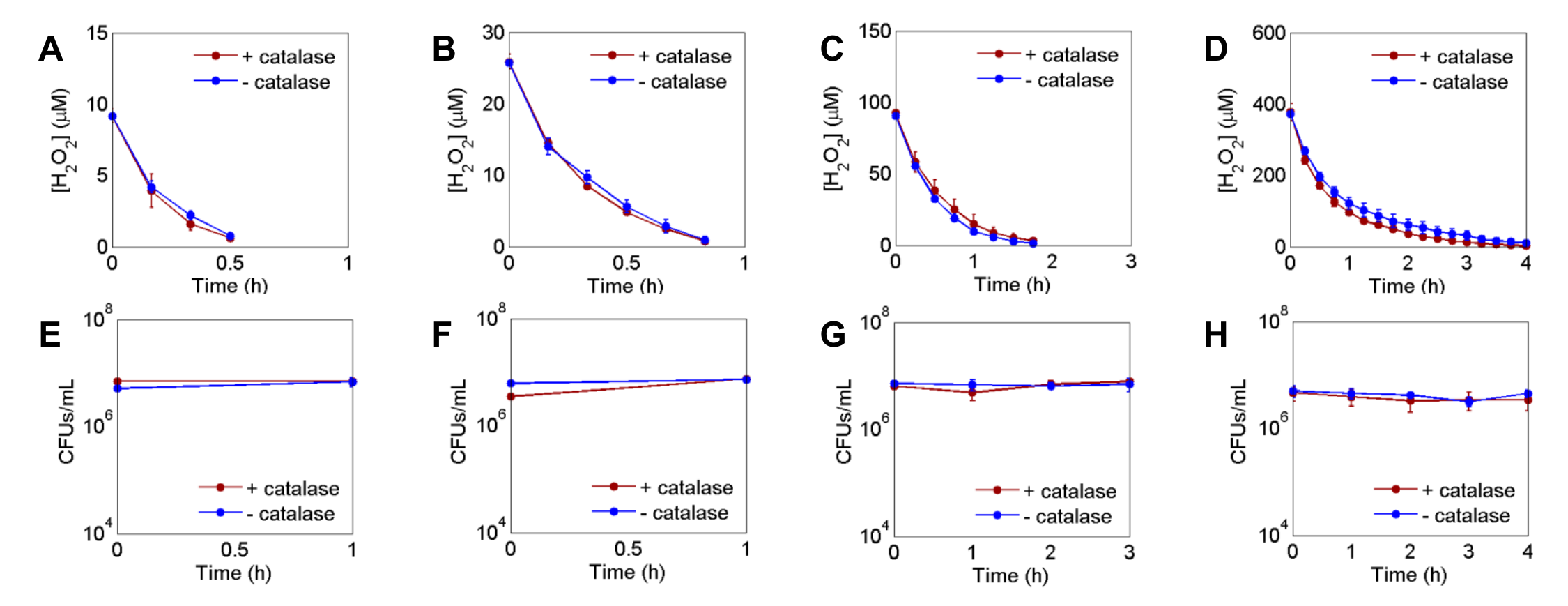

Supplement: S13 Fig — To determine whether exogenous catalase in the overnight or flask growth prior to the assay affected H2O2 clearance profiles for wild-type E. coli, we performed clearance assays in M9 media with 10 mM glucose after omitting the catalase in all pre-processing steps. A-D. H2O2 concentration did not differ significantly at any time point. E-H. CFU loss was not observed under either condition. (TIF) [file pcbi.1004562.s013.tif]

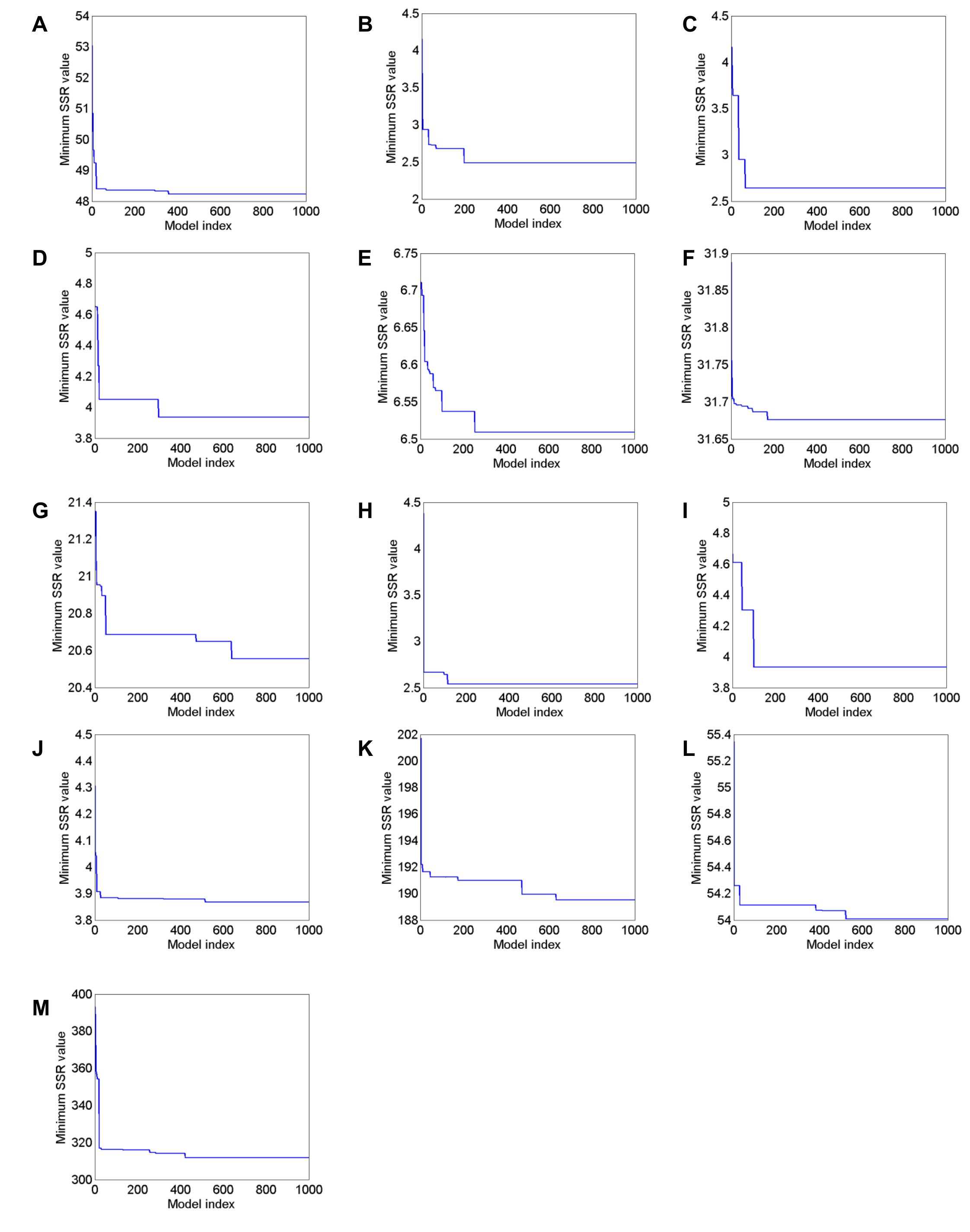

Supplement: S14 Fig — Each parameter set optimization was randomly initialized 1,000 times. Presented here is the minimum SSR that had been found at each of the 1,000 iterations (model index) for the following optimizations performed: model structure 1 (A), 2 (B), 3 (C), 4 (D), 5 (E), 6 (F), 7 (G), 8 (H), 9 (I), 10 (J) trained on wild-type data; model structure 2 (K) and 3 (L) trained on wild-type and ΔkatE ΔkatG data; model structure 3 (M) on wild-type, ΔkatE ΔkatG, ΔkatE, and ΔkatG data. This data suggested that more than 1,000 initializations would provide very little return for additional computational time invested. (TIF) [file pcbi.1004562.s014.tif]
