# Supplementary material for: A Kinetic Platform to Determine the Fate of Hydrogen Peroxide in Escherichia coli
Source: PLoS Comput Biol. 2015 Nov 6;11(11):e1004562. doi: 10.1371/journal.pcbi.1004562 (PMC4636272; doi:10.1371/journal.pcbi.1004562)
Supplement: S2 Table — All spontaneous reactions are provided with their rate constants, references, and relevant notes. Bounds are listed for uncertain parameters. (DOCX) [file pcbi.1004562.s016.docx]

Table S2: Spontaneous rate equations

| # | Reaction and rate equation | Parameters | References | Compartment |
| --- | --- | --- | --- | --- |
| 1 | •OH + O_2_•^−^ → O_2_ + OH^−^ | k=1.0 × 10^10^ M^−1^s^−1^ | [81] | Cell |
|  | r = k [•OH] [O_2_•^−^] |  |  |  |
| 2 | Met + H_2_O_2_ → Met_ox_ + H_2_O | k=6.0 x 10^-3^ M^−1^s^−1^ | [104] | Cell |
|  | r = k [Met] [H_2_O_2_] |  |  |  |
| 3 | 2 O_2_•^−^ + 2 H^+^ → O_2_ + H_2_O_2_ | k=2 x 10^5^ M^−1^s^−1^ | [111]^a^ | Cell, media |
|  | r = k [O_2_•^−^] [O_2_•^−^] |  |  |  |
| 4 | → O_2_•^−^ | k=2.9 × 10^−6^ M s^−1^ | [88]^b^ | Cell |
|  | r = k |  |  |  |
| 5 | •OH + Met → | k = 8.3 x 10^9^ M^−1^s^−1^ | [104] | Cell |
|  | r = k [•OH] [Met] |  |  |  |
| 6 | •OH + Phe → | k = 6.5 x 10^9^ M^−1^s^−1^ | [104] | Cell |
|  | r = k [•OH] [Phe] |  |  |  |
| 7 | •OH + His → | k = 1.3 x 10^10^ M^−1^s^−1^ | [104] | Cell |
|  | r = k [•OH] [His] |  |  |  |
| 8 | •OH + Ile-Leu → | k = 1.75 x 10^9^ M^−1^s^−1^ | [104]^c^ | Cell |
|  | r = k [•OH] [Ile-Leu] |  |  |  |
| 9 | •OH + Val → | k = 7.6 x 10^8^ M^−1^s^−1^ | [104] | Cell |
|  | r = k [•OH] [Val] |  |  |  |
| 10 | •OH + Pro → | k = 4.8 x 10^8^ M^−1^s^−1^ | [104] | Cell |
|  | r = k [•OH] [Pro] |  |  |  |
| 11 | •OH + Gln → | k = 5.4 x 10^8^ M^−1^s^−1^ | [104] | Cell |
|  | r = k [•OH] [Gln] |  |  |  |
| 12 | •OH + Thr → | k = 5.1 x 10^8^ M^−1^s^−1^ | [104] | Cell |
|  | r = k [•OH] [Thr] |  |  |  |
| 13 | •OH + Lys → | k = 3.4 x 10^8^ M^−1^s^−1^ | [104] | Cell |
|  | r = k [•OH] [Lys] |  |  |  |
| 14 | •OH + Ser → | k = 3.2 x 10^8^ M^−1^s^−1^ | [104] | Cell |
|  | r = k [•OH] [Ser] |  |  |  |
| 15 | •OH + Glu → | k = 2.3 x 10^8^ M^−1^s^−1^ | [104] | Cell |
|  | r = k [•OH] [Glu] |  |  |  |
| 16 | •OH + Asp → | k = 7.5 x 10^7^ M^−1^s^−1^ | [104] | Cell |
|  | r = k [•OH] [Asp] |  |  |  |
| 17 | •OH + Asn → | k = 4.9 x 10^7^ M^−1^s^−1^ | [104] | Cell |
|  | r = k [•OH] [Asn] |  |  |  |
| 18 | •OH + Arg → | k = 3.5 x 10^9^ M^−1^s^−1^ | [104] | Cell |
|  | r = k [•OH] [Arg] |  |  |  |
| 19 | •OH + Tyr → | k = 1.3 x 10^10^ M^−1^s^−1^ | [104] | Cell |
|  | r = k [•OH] [Tyr] |  |  |  |
| 20 | •OH + Trp → | k = 1.3 x 10^10^ M^−1^s^−1^ | [104] | Cell |
|  | r = k [•OH] [Trp] |  |  |  |
| 21 | •OH + Ala → | k = 7.7 x 10^7^ M^−1^s^−1^ | [104] | Cell |
|  | r = k [•OH] [Ala] |  |  |  |
| 22 | •OH + Cys → | k = 3.4 x 10^10^ M^−1^s^−1^ | [104] | Cell |
|  | r = k [•OH] [Cys] |  |  |  |
| 23 | •OH + Gly → | k = 1.7 x 10^7^ M^−1^s^−1^ | [104] | Cell |
|  | r = k [•OH] [Gly] |  |  |  |
| 24 | Fe^2+^ + H_2_O_2_ → Fe^3+^ + •OH + OH^−^ | k = 1000-50000 M^−1^s^−1^ | ^d^ | Cell |
|  | r = k [Fe^2+^] [ H_2_O_2_] |  |  |  |
| 25 | Fe^3+^ + O_2_•^−^ → Fe^2+^ + O_2_ | k = 1 x 10^8^ M^−1^s^−1^ | [105] | Cell |
|  | r = k [Fe^3+^] [ O_2_•^−^] |  |  |  |
| 26 | Fe^2+^ + O_2_•^−^ + H^+^ → Fe^3+^ + H_2_O_2_ | k = 1 x 10^7^ M^−1^s^−1^ | [106] | Cell |
|  | r = k [Fe^2+^] [ O_2_•^−^] |  |  |  |
| 27 | Fe^2+^ + •OH → Fe^3+^ + OH^−^ | k = 3.2 x 10^8^ M^−1^s^−1^ | [106] | Cell |
|  | r = k [Fe^2+^] [•OH] |  |  |  |
| 28 | Fe^2+^ + CO_3_•^−^ → Fe^3+^ + HCO_3_^−^ | k = 3.6 x 10^8^ M^−1^s^−1^ | [106] | Cell |
|  | r = k [Fe^2+^] [CO_3_•^−^] |  |  |  |
| 29 | 2 GS• → GSSG | k = 7.5 × 10^8^ M^−1^s^−1^ | [81] | Cell |
|  | r = k [GS•] [GS•] |  |  |  |
| 30 | GS• + Tyr → Tyr• + GSH | k = 3.5 × 10^5^ M^−1^s^−1^ | [81] | Cell |
|  | r = k [GS•] [Tyr ] |  |  |  |
| 31 | Tyr• + GSH → GS• + Tyr | k = 3.5 × 10^5^ M^−1^s^−1^ | [81] | Cell |
|  | r = k [GSH] [Tyr• ] |  |  |  |
| 32 | CO_3_•^−^ + GSH → HCO_3_^−^ + GS• | k = 5.3 × 10^6^ M^−1^s^−1^ | [81] | Cell |
|  | r = k [CO_3_•^−^] [GSH ] |  |  |  |
| 33 | CO_3_•^−^ + Tyr → HCO_3_^−^ + Tyr• | k = 4.5 × 10^7^ M^−1^s^−1^ | [81] | Cell |
|  | r = k [CO_3_•^−^] [Tyr ] |  |  |  |
| 34 | CO_3_•^−^ + Trp → HCO_3_^−^ + Trp• | k = 7 × 10^8^ M^−1^s^−1^ | [81] | Cell |
|  | r = k [CO_3_•^−^] [Trp ] |  |  |  |
| 35 | CO_3_•^−^ + Cys → Cys• + HCO_3_^−^ | k = 4.6 × 10^7^ M^−1^s^−1^ | [81] | Cell |
|  | r = k [CO_3_•^−^] [Cys ] |  |  |  |
| 36 | •OH + CO_3_^2−^ → CO_3_•^−^ + OH^−^ | k = 3 × 10^8^ M^−1^s^−1^ | [81] | Cell |
|  | r = k [•OH ] [CO_3_•^−^] |  |  |  |
| 37 | •OH + HCO_3_^−^ → H_2_O + CO_3_•^−^ | k = 8.5 × 10^6^ M^−1^s^−1^ | [81] | Cell |
|  | r = k [•OH ] [HCO_3_^−^] |  |  |  |
| 38 | H_2_CO_3_ → CO_2_ + H_2_O | k = 20 s^−1^ | [107] | Cell |
|  | r = k [HCO_3_^−^] |  |  |  |
| 39 | CO_2_ + H_2_O → H_2_CO_3_ | k = 0.03 s^−1^ | [107] | Cell |
|  | r = k [CO_2_] |  |  |  |
| 40 | HCO_3_^−^ + H_2_O → H_2_CO_3_ + OH^−^ | k = 0.1 s^−1^ | [107]^e^ | Cell |
|  | r = k [HCO_3_^−^] |  |  |  |
| 41 | H_2_CO_3_ + OH^−^ → HCO_3_^−^ + H2O | k = 1.0 × 10^3^ s^−1^ | [107]^f^ | Cell |
|  | r = k [H_2_CO_3_] |  |  |  |
| 42 | GSOH + GSH → GSSG + H_2_O | k = 720 M^−1^s^−1^ | [81] | Cell |
|  | r = k [GSOH] [GSH] |  |  |  |
| 43 | GSOO• → GS• + O_2_ | k = 6.2 × 10^5^ s^−1^ | [81] | Cell |
|  | r = k [GSOO•] |  |  |  |
| 44 | Trx_red_ + GSSG → Trx_ox_ + 2 GSH | k = 100 M^−1^s^−1^ | [108]^g^ | Cell |
|  | r = k [Trx_red_] [GSSG] |  |  |  |
| 45 | GS• + O_2_ → GSOO• | k = 2.0 × 10^9^ M^−1^s^−1^ | [81] | Cell |
|  | r = k [GS•] [O_2_] |  |  |  |
| 46 | GSOO• + GSH → GSO• + GSOH | k = 2 × 10^6^ M^−1^s^−1^ | [81] | Cell |
|  | r = k [GSOO •] [GSH] |  |  |  |
| 47 | GSO• + GSH → GSOH + GS• | k = 1 × 10^5^ M^−1^s^−1^ | [81] | Cell |
|  | r = k [GSO •] [GSH] |  |  |  |
| 48 | •OH + GSH → GS• + H_2_O | k = 1.4 × 10^10^ M^−1^s^−1^ | [81] | Cell |
|  | r = k [•OH][GSH] |  |  |  |
| 49 | GSSG•^−^ + O_2_ → GSSG + O_2_•^−^ | k = 5 × 10^9^ M^−1^s^−1^ | [81] | Cell |
|  | r = k [GSSG•^−^][ O_2_] |  |  |  |
| 50 | GS• + GS^−^ → GSSG•^−^ | k = 6 × 10^8^ M^−1^s^−1^ | [81] | Cell |
|  | r = k [GS•][GS^−^] |  |  |  |
| 51 | GSSG•^−^ → GS• + GS^−^ | k = 1.6 × 10^5^ s^−1^ | [81] | Cell |
|  | r = k [GSSG•^−^] |  |  |  |
| 52 | GS^−^ + H^+^ → GSH | k = 2.5 × 10^3^ s^−1^ | [85] | Cell |
|  | r = k [GS^−^] |  |  |  |
| 53 | GSH → GS^−^ + H^+^ | k = 63.1 s^−1^ | [85] | Cell |
|  | r = k [GSH] |  |  |  |
| 54 | GS^−^ + CO_3_•^−^ → GS• + CO_3_^2−^ | k = 7.1 × 10^8^ M^−1^s^−1^ | [85] | Cell |
|  | r = k [GS^−^] [CO_3_•^−^] |  |  |  |
| 55 | O_2_•^−^ + GSH + H^+^ → GS• + H_2_O_2_ | k = 200 M^−1^s^−1^ | [81] | Cell |
|  | r = k [O_2_•^−^] [GSH] |  |  |  |
| 56 | H_2_O_2_ → H_2_O + 0.5 O_2_ | k = 0 - 0.0331 h^-1^ | ^h^ | Cell, media |
|  | r = k [H_2_O_2_] |  |  |  |
| 57 | Pyr + H_2_O_2_ → Ace + CO_2_ + H_2_O | k = 2.3 M^−1^s^−1^ | ^i^ | Cell |
|  | r = k [H_2_O_2_] [Pyr] |  |  |  |
| 58 | protein → | k = 8.36 × 10^-6^ - 9.63 × 10^-3^ s^−1^ | ^j^ | Cell |
|  | r = k [protein] |  |  |  |
| 59 | protein + H_2_O_2_ → | k = 8.5 × 10^-5^ - 14.3 M^−1^s^−1^ | ^k^ | Cell |
|  | r = k [H_2_O_2_] [protein] |  |  |  |
| 60^l^ | HPI + H_2_O_2_ $\underset{\to}{HPI deactivation}$ | $k_{\deg}$  $K_{H_{2}O_{2}}=4.2\times{10}^{-3}M$ | *  [91]^m^ | Cell |
|  | $r=\frac{k_{\deg}\left[ H_{2}O_{2} \right][HPI]}{\left[ H_{2}O_{2} \right]+K_{H_{2}O_{2}}}$ |  |  |  |
| 61^l^ | HPII + H_2_O_2_ $\underset{\to}{HPII deactivation}$ | $k_{d}$  $K_{H_{2}O_{2}}=2\times{10}^{-2}M$ | *  [92]^m^ | Cell |
|  | $r=\frac{k_{\deg}\left[ H_{2}O_{2} \right][HPII]}{\left[ H_{2}O_{2} \right]+K_{H_{2}O_{2}}}$ |  |  |  |
| 62 | O_2,air_ $\underset{\to}{O_{2,trans}}$ O_2,culture_ | $k_{L}a_{O_{2}}=0.0164 s^{-1}$ | [41]^n^ | Total |
|  | $r=k_{L}a_{O_{2}}({{[O}_{2}]}_{\mathrm{sat}}-\left[ O_{2} \right])$ |  |  |  |
| 63 | H_2_O_2,media_ $\underset{\to}{{H_{2}O}_{2,trans}}$ H_2_O_2,cell_ | $k_{c,eff}$ | *^o^ | Total |
|  | $r=k_{c,eff}({{[H_{2}O}_{2}]}_{\mathrm{ex}}-\left[ {H_{2}O}_{2} \right]_{\mathrm{int}})$ |  |  |  |

* indicates uncertain parameters that were optimized during the training procedure.

The concentrations of the following metabolites were held constant: Met, Phe, His, Ile-Leu, Val, Pro, Gln, Thr, Lys, Ser, Glu, Asp, Asn, Arg, Tyr, Trp, Ala, Cys, Gly, Pyr, Ace, and O_2,air_.

^a^ Rate for spontaneous superoxide dismutation at pH 7.8

^b^ Superoxide production in *E. coli* in minimal medium

^c^ Rate constants averaged for leucine and isoleucine because individual amino acid concentrations were not available.

^d^ Rate varies from 1000 to 50000 M^-1^s^-1^[21] (3.6-180 uM^-1^h^-1^). It is varied in each optimization, and can be different in each parameter set.

^e^ Calculated for pH 7.6.

^f^ "Instantaneous."

^g^ Measured at 25°C and pH 7.5.

^h^ Parameter fit using cell-free controls: 0.0324 h^-1^ for M9 10 mM glucose, 0.0331 h^-1^ for M9 10 mM glucose with 100 μg/mL CAM, and 0 h^-1^ for M9 lacking glucose.

^i^ Rate constant obtained by extracting values from plot in [109] and fitting with "WebPlotDigitizer" online tool.

^j^ This rate varies with each parameter set when included in the optimization. Some model types do not have a constant degradation reaction. In the models that use the "general" protein degradation rate reported previously, the rate is 2.8 × 10-5 s^-1^ [34]. The optimized parameter could vary between the lowest degradation rate (half-life > 23 h [35]) and the fastest degradation rate (half-life = 1.2 min [36]) we found in the literature.

^k^ These rates vary with each parameter set, and the reaction does not appear in the constant degradation models. Degradation rates reported for Aspergillus niger and bovine catalase were 0.0085 and 0.153 M^−1^s^−1^ [40], respectively. Due to major differences in experimental setup and the organism used for [40] and this paper, this parameter was varied two orders of magnitude lower than the lowest reported value, and two higher than the highest reported value. The same range was used for AHP because no other information was available. There is no information available regarding *E. coli* AhpC, and inactivation kinetics vary greatly between organisms [39].

^l^ Catalase is inactivated by excess substrate. The inactivation was best described by the rate equation used for the catalytic reaction, with k_cat_ replaced with a deactivation constant k_deg_. Deactivation rate constants varied from 0.46$\times$10^-4^ (*Aspergillus niger*) to 6.8 $\times$10^-4^  s^-1^ (bovine catalase) [37]. Because *Aspergillus* and bovine parameters can be expected to be distinct from *E. coli*, and over an order of magnitude difference was seen between these two species, we allowed the degradation parameter to be two orders of magnitude lower than *Aspergillus* and two orders of magnitude higher than that of bovine catalase. The bounds on this parameter during optimization were 0.46$\times$10^-6^  to 6.8 $\times$10^-2^  s^-1^.

^m^ The K_M_ for the deactivation will be equal to that of the substrate binding to the enzyme [92].

^n^ The mass transfer coefficient was measured in an open baffled shake flask at 37°C shaking at 250 rpm [110].

^o^ Maximum and minimum bounds of the effective mass transfer coefficient were based on the permeability coefficient of H_2_O_2_ across cell membranes, which was measured by Seaver and Imlay [41] as 1.6 x 10^-3^  cm s^-1^. Using the cellular surface area and cell density of our system, the permeability coefficient translates into a k_c,eff_ of 0.25 s^-1^. Therefore, the minimum bound for k_c,eff_ was set at this value, and the maximum was set two orders of magnitude higher to account for the turbulent mixing of the shake flask system used in this study.

Note: References are listed in the main text.
