# Supplementary material for: A Kinetic Platform to Determine the Fate of Hydrogen Peroxide in Escherichia coli
Source: PLoS Comput Biol. 2015 Nov 6;11(11):e1004562. doi: 10.1371/journal.pcbi.1004562 (PMC4636272; doi:10.1371/journal.pcbi.1004562)
Supplement: S3 Table — All enzymatic reactions are provided with their rate equations, constants, and references. Bounds are listed for uncertain parameters, which are indicated by an asterisk. (DOCX) [file pcbi.1004562.s017.docx]

Table S3: Enzymatic rate equations

| # | Reaction and rate equation | Parameters | Refs. |
| --- | --- | --- | --- |
| 64 | H_2_O + Trx_red_ + Met_ox_ $\underset{\to}{\mathrm{Msr}}$ Trx_ox_ + Met | $k_{\mathrm{cat}}=3.7 s^{-1}$  $K_{\mathrm{Trx}_{\mathrm{red}}}={10}^{-5}M$  $K_{\mathrm{Met}_{\mathrm{ox}}}=1.9\times{10}^{-3}M$ | [112]  [112]  [112] |
|  | $r=\frac{k_{\mathrm{cat}}\left[ \mathrm{Met}_{\mathrm{ox}} \right]\left[ \mathrm{Trx}_{\mathrm{red}} \right][Msr]}{K_{\mathrm{Trx}_{\mathrm{red}}}\left[ \mathrm{Met}_{\mathrm{ox}} \right]+K_{\mathrm{Met}_{\mathrm{ox}}}\left[ \mathrm{Trx}_{\mathrm{red}} \right]+\left[ \mathrm{Met}_{\mathrm{ox}} \right][\mathrm{Trx}_{\mathrm{red}}]}$ |  |  |
| 65 | H_2_O_2_ + Trx_red_ $\underset{\to}{\mathrm{Tpx}}$ Trx_ox_ + 2 H_2_O | $k_{\mathrm{cat}}=76 s^{-1}$  $K_{\mathrm{Trx}_{\mathrm{red}}}=2.55\times{10}^{-5}M$  $K_{H_{2}O_{2}}=1.73\times{10}^{-3}M$ | [113]  [113]  [113] |
|  | $r=\frac{k_{\mathrm{cat}}\left[ H_{2}O_{2} \right]\left[ \mathrm{Trx}_{\mathrm{red}} \right][Tpx]}{K_{\mathrm{Trx}_{\mathrm{red}}}\left[ H_{2}O_{2} \right]+K_{H_{2}O_{2}}\left[ \mathrm{Trx}_{\mathrm{red}} \right]+\left[ H_{2}O_{2} \right][\mathrm{Trx}_{\mathrm{red}}]}$ |  |  |
| 66 | H_2_O_2_ + Trx_red_ $\underset{\to}{\mathrm{Bcp}}$ Trx_ox_ + 2 H_2_O | $k_{\mathrm{cat}}=1.24 s^{-1}$  $K_{\mathrm{Trx}_{\mathrm{red}}}=5\times{10}^{-4}M$  $K_{H_{2}O_{2}}=7.61\times{10}^{-5}M$ | [114]  [114]  [114] |
|  | $r=\frac{k_{\mathrm{cat}}\left[ H_{2}O_{2} \right]\left[ \mathrm{Trx}_{\mathrm{red}} \right][Bcp]}{K_{\mathrm{Trx}_{\mathrm{red}}}\left[ H_{2}O_{2} \right]+K_{H_{2}O_{2}}\left[ \mathrm{Trx}_{\mathrm{red}} \right]+\left[ H_{2}O_{2} \right][\mathrm{Trx}_{\mathrm{red}}]}$ |  |  |
| 67 | $\underset{\to}{\mathrm{HPI}_{\exp}}$HPI | $k_{HPI-exp,max}$  $K_{{HPI-exp,H}_{2}O_{2}}$ | *^a^  *^b^ |
|  | $r=\frac{k_{HPI-exp,max}\left[ H_{2}O_{2} \right]}{\left[ H_{2}O_{2} \right]+K_{{HPI-exp,H}_{2}O_{2}}}$ |  |  |
| 68 | $\underset{\to}{\mathrm{AHP}_{\exp}}$Ahp | $k_{AHP-exp,max}$  $K_{{AHP-exp,H}_{2}O_{2}}$ | *^a^  *^b^ |
|  | $r=\frac{k_{AHP-exp,max}\left[ H_{2}O_{2} \right]}{\left[ H_{2}O_{2} \right]+K_{{AHP-exp,H}_{2}O_{2}}}$ |  |  |
| 69 | 2 H_2_O_2_ $\underset{\to}{\mathrm{HPI}}$ 2 H_2_O + O_2_ | $k_{\mathrm{cat}}=2950 s^{-1}$  $K_{H_{2}O_{2}}=4.2\times{10}^{-3}M$ | [91]  [91] |
|  | $r=\frac{k_{\mathrm{cat}}\left[ H_{2}O_{2} \right][HPI]}{\left[ H_{2}O_{2} \right]+K_{H_{2}O_{2}}}$ |  |  |
| 70 | 2 H_2_O_2_ $\underset{\to}{\mathrm{HPII}}$ 2 H_2_O + O_2_ | $k_{\mathrm{cat}}=26,700 s^{-1}$  $K_{H_{2}O_{2}}=2\times{10}^{-2}M$ | [92]  [92] |
|  | $r=\frac{k_{\mathrm{cat}}\left[ H_{2}O_{2} \right][HPII]}{\left[ H_{2}O_{2} \right]+K_{H_{2}O_{2}}}$ |  |  |
| 71 | NADH + H^+^ + H_2_O_2_ $\underset{\to}{\mathrm{AHP}}$ NAD^+^ + 2 H_2_O | $k_{\mathrm{cat}}=52.4 s^{-1}$  $K_{\mathrm{NADH}}$=$1.41\times{10}^{-6}M$  $K_{H_{2}O_{2}}=1.4\times{10}^{-6}M$ | [38]^c^  [94]^c^  [38]^c^ |
|  | $r=\frac{k_{\mathrm{cat}}\left[ H_{2}O_{2} \right]\left[ \mathrm{NADH} \right][AHP]}{K_{\mathrm{NADH}}\left[ H_{2}O_{2} \right]+K_{H_{2}O_{2}}\left[ \mathrm{NADH} \right]+\left[ H_{2}O_{2} \right][NADH]}$ |  |  |
| 72 | Trx_ox_ + NADPH + H^+^ $\underset{\to}{\mathrm{TrxR}}$ Trx_red_ + NADP^+^ | $k_{\mathrm{cat}}=41.25 s^{-1}$  $K_{\mathrm{Trx}_{\mathrm{ox}}}$=$4.6\times{10}^{-6}M$  $K_{\mathrm{NADPH}}$=$1.7\times{10}^{-6}M$ | [115]  [115]  [115] |
|  | $r=\frac{k_{\mathrm{cat}}\left[ \mathrm{Trx}_{\mathrm{ox}} \right]\left[ \mathrm{NADPH} \right][TrxR]}{K_{\mathrm{Trx}_{\mathrm{ox}}}\left[ \mathrm{NADPH} \right]+K_{\mathrm{NADPH}}\left[ \mathrm{Trx}_{\mathrm{ox}} \right]+\left[ \mathrm{NADPH} \right][\mathrm{Trx}_{\mathrm{ox}}]}$ |  |  |
| 73^d^ | GSSG + H^+^ + NADPH $\underset{\to}{\mathrm{Gor}}$ 2 GSH + NADP^+^ | $k_{1}=267 s^{-1}$  $k_{2}=6.55\times{10}^{5}M^{-1} s^{-1}$  $K_{\mathrm{NADPH}}=2.2\times{10}^{-5}M$  $K_{\mathrm{GSSG}}=9.7\times{10}^{-5}M$  $K_{1}=0.022$  $K_{2}=3.9\times{10}^{3}M^{-1}$ | [116]  [117]^e^  [116]  [116]  [117]^e^  [117]^e^ |
|  | $r=\frac{k_{1}\left[ \mathrm{GSSG} \right]\left[ \mathrm{NADPH} \right][Gor]+k_{2}\left[ \mathrm{GSSG} \right]^{2}[NADPH][Gor]}{K_{\mathrm{NADPH}}\left[ \mathrm{GSSG} \right]+K_{\mathrm{GSSG}}\left[ \mathrm{NADPH} \right]+\left[ \mathrm{GSSG} \right]\left[ \mathrm{NADPH} \right]+ K_{1}{[GSSG]}^{2}{+K}_{2}{[GSSG]}^{2}[NADPH]}$ |  |  |
| 74 | 2 O_2_•^−^ + 2 H^+^ $\underset{\to}{\mathrm{MnSOD}}$ O_2_ + H_2_O_2_ | k = 6.8 x 10^8^ M^−1^s^−1^ | [111] |
|  | r = k [O_2_•^−^] [MnSOD] |  |  |
| 75 | 2 O_2_•^−^ + 2 H^+^ $\underset{\to}{\mathrm{FeSOD}}$ O_2_ + H_2_O_2_ | k = 6.6 x 10^8^ M^−1^s^−1^ | [111] |
|  | r = k [O_2_•^−^] [FeSOD] |  |  |

* indicates uncertain parameters that were optimized during the training procedure.

The concentrations of the following metabolites were held constant: Met, NADH, NAD+, NADPH, and NADP+.

All enzymatic reactions take place in the intracellular compartment.

^a^ Parameters were varied from 2 $\times$10^-6^ to 2$\times$10^-8^ M s^-1^; see explanation in [33].

^b^ Bounds on K_AHP-exp,H2O2_ and K_HPI-exp,H2O2_ were approximated by the work of Kotte and colleagues [34], which varied from approximately 2 nM to 1 mM.

^c^ Kinetic parameters were not available for *E. coli*. The values presented are from *S.* Typhimurium, which also uses AhpC as the primary scavenger of endogenously produced H_2_O_2_. We found 98% protein sequence identity for AhpC and 95% identity for AhpF when *E. coli* MG1655 protein sequences were blasted against those for *S.* Typhimurium.

^d^ Form of rate expression from [117].

^e^ Kinetic parameters were not available for *E. coli*. The values presented are from glutathione reductase in rat liver.

Note: References are listed in the main text.
