# Supplementary material for: A Kinetic Platform to Determine the Fate of Hydrogen Peroxide in Escherichia coli
Source: PLoS Comput Biol. 2015 Nov 6;11(11):e1004562. doi: 10.1371/journal.pcbi.1004562 (PMC4636272; doi:10.1371/journal.pcbi.1004562)
Supplement: S4 Table — (DOCX) [file pcbi.1004562.s018.docx]

| Deletion | Forward external primer | Reverse external primer | Forward internal primer | Reverse internal primer |
| --- | --- | --- | --- | --- |
| Δ*katE* | 5’- TATTTGCCACGCAGCATCCA-3’ | 5’- TTGAGACTGCTGACAAACGCAAA-3’ | 5’- aaaaactcaccggacgtgac-3’ | 5’- tcacccattcgggagtagag-3’ |
| Δ*katG* | 5’- ATCTCAACTATCGCATCCGTGGA-3’ | 5’- CAGGGCAATGGCTAAGGTGTATTTA-3’ | 5’- caaccgagatgggtctgatt-3’ | 5’- ctcaacaccaaccacaccag-3’ |
| Δ*ahpCF* | 5’- AAGATACCAAAGGGTAGTTCAGATTA-3’ | 5’- TAAGTATCCCGCCCTGCCCG-3’ | 5’- tcggcagaaatcaaggaact-3’ | 5’- cagtaggtcacgcctttggt-3’ |

The following internal kanR reverse primer was used with the forward primers external to genes to check for proper chromosomal integration: 5’-ATGATGGATACTTTCTCGGCAGGAG-3’
